# Multiscale modelling of blood flow in cerebral microcirculation: Details at capillary scale control accuracy at the level of the cortex

**DOI:** 10.1371/journal.pone.0189474

**Published:** 2018-01-11

**Authors:** Myriam Peyrounette, Yohan Davit, Michel Quintard, Sylvie Lorthois

**Affiliations:** 1 Institut de Mécanique des Fluides de Toulouse, IMFT, Université de Toulouse, CNRS - Toulouse, France; 2 Nancy E. and Peter C. Meinig School of Biomedical Engineering, Cornell University, Ithaca, NY, United States of America; Fraunhofer Research Institution of Marine Biotechnology, GERMANY

## Abstract

Aging or cerebral diseases may induce architectural modifications in human brain microvascular networks, such as capillary rarefaction. Such modifications limit blood and oxygen supply to the cortex, possibly resulting in energy failure and neuronal death. Modelling is key in understanding how these architectural modifications affect blood flow and mass transfers in such complex networks. However, the huge number of vessels in the human brain—tens of billions—prevents any modelling approach with an explicit architectural representation down to the scale of the capillaries. Here, we introduce a hybrid approach to model blood flow at larger scale in the brain microcirculation, based on its multiscale architecture. The capillary bed, which is a space-filling network, is treated as a porous medium and modelled using a homogenized continuum approach. The larger arteriolar and venular trees, which cannot be homogenized because of their fractal-like nature, are treated as a network of interconnected tubes with a detailed representation of their spatial organization. The main contribution of this work is to devise a proper coupling model at the interface between these two components. This model is based on analytical approximations of the pressure field that capture the strong pressure gradients building up in the capillaries connected to arterioles or venules. We evaluate the accuracy of this model for both very simple architectures with one arteriole and/or one venule and for more complex ones, with anatomically realistic tree-like vessels displaying a large number of coupling sites. We show that the hybrid model is very accurate in describing blood flow at large scales and further yields a significant computational gain by comparison with a classical network approach. It is therefore an important step towards large scale simulations of cerebral blood flow and lays the groundwork for introducing additional levels of complexity in the future.

## 1 Introduction

**Fig 1:**
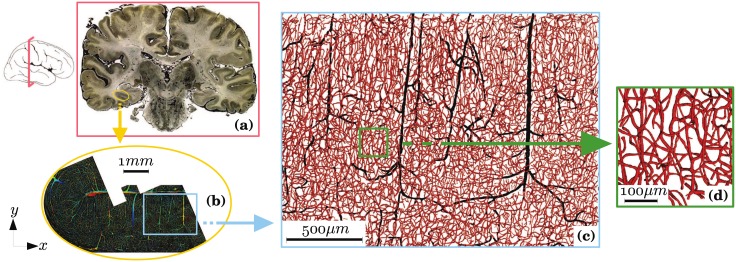
Multiscale architecture of microvascular networks in human brain with a representation of different length scales. **(a)** *Brain scale (∼63 cm^2^ × 300 μm)*: 300 *μ*m-thick cortical section, where blood vessels have been injected with India ink for contrast enhancement [7]. **(b)** *Macroscopic scale (∼18 mm^2^ × 300 μm)*: reconstruction of parts of the collateral sulcus by confocal laser microscopy [8]. **(c)** *Mesoscopic scale (∼5 mm^2^ × 300 μm)*: region of interest in which vessels of more than 10 *μ*m in diameter are colored in black and vessels of less than 10 *μ*m in diameter are colored in red (diameters have been multiplied by 2 for visualization). In contrast with the capillary bed, the arteriolar and venular trees have a quasi-fractal structure [9]. **(d)** *Microscopic scale (∼0.07 mm^2^ × 300 μm)*: detailed view of the capillary bed. The capillary bed is dense and space-filling over a cut-off length of ∼ 50 *μ*m [9].

Blood flow through cerebral microcirculation is the primary driver of oxygen transport and waste removal in the cortex, making microcirculation fundamental to cerebral physiology [1–3]. Yet microvascular networks have been given much less emphasis than other fascinating components of the brain, such as the white matter and the activity of neural networks [4]. Thus, we comparatively understand little about the structure and functions of these networks, let alone their regulation mechanisms, robustness to perturbations and involvement into pathologies. For instance, obstructions of small vessels during strokes, long-term capillary rarefaction or lymphocyte stalling occurring early in Alzheimer’s Disease (AD) [5] may limit the transport of oxygen to the cerebral tissue and result in energy failure and neuronal death. Understanding how these disease-induced modifications of the network architecture affect transport mechanisms in the brain is therefore a fundamental challenge that may ultimately lead to clinical breakthroughs. One of the most complex aspect of the problem is that it involves a broad range of spatial scales [6], ranging from the scale of the whole brain (∼10 cm, see Fig 1**(a)**) to the microscopic scale that we characterize here by the length of a capillary vessel *l*_*cap*_ (∼50 *μ*m, see Fig 1**(d)**), with an intermediate macroscopic scale corresponding to the thickness of the cortex (∼3 mm, see Fig 1**(b)**). This multiscale architecture makes it difficult not only to understand the mechanisms occurring at the different levels, but also to understand and assess the systemic impact of localized effects.

Advances in both imaging techniques and modelling approaches have made progress possible in the last decade. In particular, multiphoton laser scanning microscopy [10, 11], a cutting-edge fluorescence microscopy with unprecedented microscopic scale resolution and cortical penetration depth, has enabled the spatio-temporal investigation of hemodynamics and mass transfers at macroscopic scale in the living brain of healthy or diseased rodents (e.g. [1, 3, 12, 13]). In parallel, hemodynamically-based perfusion techniques are widely used in clinics to explore much larger volumes of human brain, typically the scale of the whole brain, with a resolution of approximately 10-100 mm^3^. With regard to imaging techniques, the investigation of the whole range of scales, from the microscopic to the brain scale, is therefore possible. For models, the most successful approaches are network representations that have been developed to simulate blood flow and mass transport in brain microcirculation [14–17]. However, contrary to imaging techniques, the flow models available do not cover the whole spectrum of spatial scales and simulations of transport mechanisms in the brain cannot yet be performed at clinically relevant scales.

To understand better the limitations of network models, we first need to describe them in more detail. Such approaches treat the vasculature as a network of interconnected tubes. The advantage of doing so is that the flow equations do not have to be computed over a complex three-dimensional geometry, as the relationship between average blood flow and pressure drop in each tube can be described analytically. This simplification of the geometry greatly reduces the computational cost, often yielding a simple linear system with relatively fewer variables compared to a complete three-dimensional resolution of the flow. The geometry and topology of the vascular network can be either extracted from available anatomical databases (1-10 mm^3^) or synthetically generated to investigate larger volumes, as was proposed in [18, 19]. Non-linearities induced by the complex rheology of blood in individual vessels (e.g. Fåhræus, Fåhræus-Lindqvist, and phase separation effects) can also be taken into account using empirical laws, and solved using iterative methods [20]. The main limitation of this framework is that the computational cost of the simulations quickly grows with the number of vessels. This is because, although it simplifies the geometry, the approach remains a microscopic scale description of the flow. Thus, simulations become intractable in large microvascular networks, especially when performing many statistical realizations (e.g. for studying the effect of capillary occlusions), when needing thousands of iterations to accurately capture non-linear effects, or when coupling blood flow with oxygen transport. To the best of our knowledge, the most advanced computations have reached a volume of about 30 mm^3^ (∼250 000 vessels, [18]) so far, a number that is several orders of magnitude lower than what would be necessary for brain scale simulations.

Can we develop models with the potential to simulate transport at the scale of the whole brain? The simplest way to proceed would be to use homogenized descriptions whereby the velocity and pressure fields are averaged in space over a large number of vessels and the detail of the architecture at microscopic scale is not fully described. These microscopic details are implicitly represented via effective parameters (e.g. permeability) that characterize the flow at a larger scale, which we term mesoscopic scale (Fig 1**(c)**). Such approaches, which we term “homogenized” or “continuum approaches”, are prominent in porous media sciences [21–23], the most famous example being Darcy’s law. One of the advantages of such descriptions is that they lend themselves very well to the assimilation of experimental or numerical data obtained in relatively small volumes. For example, the permeability can be obtained by numerical simulations at microscopic scale using anatomical data acquired in only ~ 1 mm^3^ [14, 24]. This class of approaches also significantly reduces the computational cost compared to network models, as computations are performed over coarse mesoscopic scale grids. Unfortunately, homogenized models are not applicable to the flow problem in the whole brain microvasculature. The primary reason is that simple average representations are only available when the structure exhibits a discrete hierarchy of scales, not a continuous one as the brain microvasculature (Fig 1). This continuous hierarchy of scales stems from the architecture of feeding arteriolar and draining venular trees, which exhibit a quasi-fractal geometry [9] (highlighted in black in Fig 1**(c)**).

**Fig 2:**
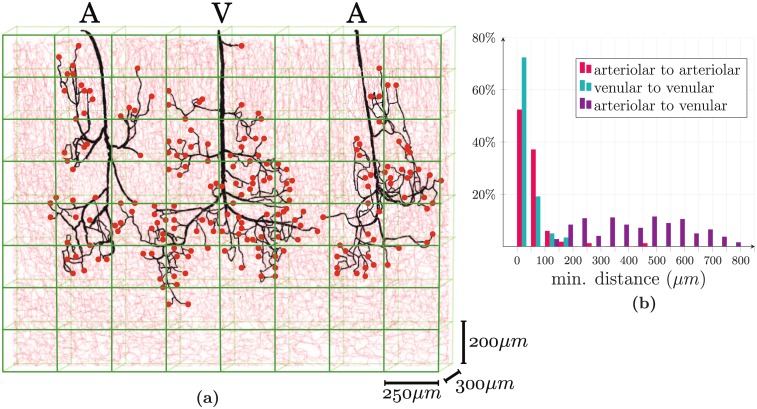
Schematic diagrams of the hybrid approach and spatial correlations between coupling sites. **(a)** The arteriolar (A) and venular (V) trees (black networks) plunge into the cortical tissue where their endpoints are connected to the capillary bed (light red). Because of the multiscale architecture of the human brain (Fig 1), we adopt two separate strategies to model blood flow at macroscopic scale, depending on the hierarchical position of the vessels in the microvascular network. A network approach is used in arteriolar and venular trees while the capillary bed is considered as a continuum. The continuum model is then discretized using coarse mesh cells (green) at mesoscopic scale, with size *h_x_* × *h_y_* × *h_z_* ≃ (250 *μ*m)^3^. The side of a discretization cell is significantly larger than the capillary characteristic length *l_cap_* = 50 *μ*m. The red dots correspond to the coupling points connecting arterioles (or venules) to capillary vessels, where coupling conditions must be applied. **(b)** We use anatomically accurate data to illustrate spatial correlations between the coupling sites. Minimum distances have been computed between all coupling points according to their types (arteriolar to arteriolar, venular to venular and arteriolar to venular). The histogram plot presents the percentages of couplings associated to the same range of minimum distance.

To bridge the gap, new modelling paradigms are needed. One such paradigm is a hybrid approach where the capillary bed (highlighted in red in Fig 1**(c)** and 1**(d)**), which is homogeneous and space-filling above a cut-off length of ∼50 *μ*m [9], is described using an averaged description, whereas feeding arteriolar and draining venular trees (highlighted in black in Fig 1**(c)**) are treated using the classical network approach. The term “hybrid” is a reference to the fact that this model is a hybrid between network and homogenized approaches. Such methods have already been introduced in the context of cardiac [25, 26] or tumor [27] microcirculation. A major difficulty of such descriptions is to properly couple both frameworks, an issue that has not been addressed in these previous papers. The coupling is difficult primarily because variables that are seemingly identical do not really have the same physical meaning. For instance, the blood pressure in the continuum approach represents a field that is spatially averaged at mesoscopic scale, while the pressure of the network approach represents a cross-sectional average within each tube. Simply imposing a continuity of the pressures at each coupling point, i.e., at each connection of an arteriolar or venular vessel to a capillary (red dots in Fig 2**(a)**), may therefore not accurately describe the strong gradients that occur locally and important errors could propagate through the whole system. Similar issues apply for concentrations or temperatures when molecular exchanges or heat transfers are considered.

In this paper, our goal is to devise a theory for coupling network and continuum descriptions of blood flow in the brain microcirculation, which lays the groundwork for modelling microvascular blood flow at the scale of the whole brain and extending to mass transfers. We focus here on blood flow with the following main simplifications: the capillary bed is assumed to be isotropic, homogeneous and its architecture is represented by simplified three-dimensional lattices, in the same spirit as in the two-dimensional study in [14]; further, the non-linearities induced by red blood cell partitioning at microvascular bifurcations are neglected, following [1, 14, 16, 18, 28], so that the hematocrit is uniform. Based on these simplifications, we introduce an analytical model describing the coupling between the network (Section 2.2) and the continuum (Section 2.3) approaches. This model takes the form of a function linking the discrete pressures at the arteriolar and venular coupled endpoints to the cell pressures in a finite volume formulation of the homogenized equations. To derive this model (Section 2.4), we use a decomposition of the pressure field around each coupling point into two analytical parts: a linear variation that captures the local network structure of the underlying capillaries close to the coupling point and a spherical solution of the continuum equations further away. Our method is validated by comparing simulations obtained using this hybrid approach to a complete network description where all the vessels including the capillaries are described by the network approach. We first assess the robustness of the approach in various basic situations, with a small number (< 2) of coupling points (Section 3.1). We then go on to consider anatomically accurate arteriolar and venular trees that interact through many coupling points with an idealized capillary bed (Section 3.2). Besides a significant improvement in computational performance (Section 3.3), our results show that properly coupling the different modelling components in the hybrid approach—not just continuity of pressures at the coupling points—is crucial to derive accurate representations of pressure and velocity fields in the cerebral microcirculation. By thoroughly exploring the theoretical framework in model situations, we lay the groundwork for the development of more general models accounting for additional levels of complexity in the future.

## 2 Models and method

Here, we present the models describing mass and momentum transport in the microcirculation for the network approach (black arteriolar and venular trees in Fig 2), the continuum representation (coarse green mesh in Fig 2) that replaces the capillary bed (light red vessels in Fig 2) and the coupling conditions at the interface between these two frameworks. For consistency, the colors conventions introduced in Fig 2 are used throughout (red dots in Fig 2).

### 2.1 Main simplifications and hypotheses

The simplifications mentioned in the Introduction (isotropy and homogeneity of the capillary bed at mesoscopic scales, uniform hematocrit) are needed to introduce the analytical coupling model presented in detail in Section 2.4. In addition to these, we use several other hypotheses that are common to all frameworks. First, blood typically flows at about 10 mm · s^-1^ in the arteriolar and venular trees and at about 1 mm · s^-1^ in the capillary network, so that the Reynolds number is everywhere significantly lower than 1 [29] and inertial effects can be neglected. Second, the pulsatility is negligible because the Womersley number is lower than 0.1 [30]. We further assume that the mass density of blood *ρ* and the gravity vector ***g*** are constant in the brain microcirculation, so that the pressures used in all equations are defined as the fluid pressures minus the hydrostatic pressures.

### 2.2 Network approach for arteriolar and venular trees

The arteriolar and venular vessels are treated as a network of interconnected tubes where blood flow is described by a succession of linear relationships between the flow rate and the pressure drop [1, 14–16, 18, 20, 31]. The flow *q*_*αβ*_ in each vessel connecting vertices *α* and *β* is written as

$$\begin{array}{c} q_{\alpha \beta }=G_{\alpha \beta }(\pi_{\alpha }-\pi_{\beta }), \end{array}$$ (1)

with *π*_*α*_, *π*_*β*_ the pressures at the vertices and *G*_*αβ*_ the vessel conductance defined as

$$\begin{array}{c} G_{\alpha \beta }=\frac{\pi d_{\alpha \beta }^{4}}{128\;\mu_{\alpha \beta }^{\text{app}}\;l_{\alpha \beta }}, \end{array}$$ (2)

with *d*_*αβ*_ the vessel mean diameter and *l*_*αβ*_ the vessel length. The apparent viscosity $\mu_{\alpha \beta }^{\text{app}}$ accounts for the emergence of complex microvascular flow structures (e.g. cell free layer) an their impact on average viscous dissipation at vessel scale [32]. In other words, this effective property accounts for the Fåhræus–Lindqvist effect [33], i.e., the dependence of viscosity upon vessel diameter *d*_*αβ*_ and discharge hematocrit *H*_*αβ*_. Although theoretical approaches can be used to derive expressions for the apparent viscosity (e.g. [34, 35]), we use the *in* *vivo* viscosity law derived by Pries *et* *al*. in [36],

$$\begin{array}{c} \mu_{\alpha \beta }^{\text{app}}=\mu_{\text{p}}\{1+\left[\mu_{\alpha \beta }^{0.45}-1\right]\frac{{\left[1-H_{\alpha \beta }\right]}^{C}-1}{{\left[1-0.45\right]}^{C}-1}\\ \times {\left[\frac{d_{\alpha \beta }}{d_{\alpha \beta }-1.1}\right]}^{2}\}{\left(\frac{d_{\alpha \beta }}{d_{\alpha \beta }-1.1}\right)}^{2}, \end{array}$$ (3)

where *μ*_p_ represents the viscosity of the plasma, $\mu_{\alpha \beta }^{0.45}$ is the apparent viscosity of blood for a discharge hematocrit of 0.45

$$\begin{array}{c} \mu_{\alpha \beta }^{0.45}=6e^{-0.085d_{\alpha \beta }}+3.2-2.44e^{-0.06d_{\alpha \beta }^{0.645}} \end{array}$$ (4)

and *C* is a coefficient describing the dependence upon hematocrit

$$\begin{array}{cc} C=(0.8+e^{-0.075d_{\alpha \beta }}) & \left(-1+\frac{1}{1+10^{-11}d_{\alpha \beta }^{12}}\right)\\ & +\frac{1}{1+10^{-11}d_{\alpha \beta }^{12}}. \end{array}$$ (5)

Further, mass balance at each inner vertex *α*—a vertex where no boundary condition is imposed—reads

$$\begin{array}{c} \sum_{\beta \in N_{\alpha ,\text{in}}}G_{\alpha \beta }(\pi_{\alpha }-\pi_{\beta })=q_{s}, \end{array}$$ (6)

where $N_{\alpha ,\text{in}}$ represents the set of inner neighbouring vertices that are connected to *α* and *q*_*s*_ is a flow rate source term that can either describe boundary conditions for arteriolar inlets (*q*_*s*_ > 0) and venular outlets (*q*_*s*_ < 0) or coupling conditions for vertices connected to the homogenized capillary bed (red dots in Fig 2**(a)**). This latter case is detailed in Section 2.4. Alternately, when a pressure condition *π*_*s*_ is imposed on a boundary, the corresponding vertex is called an outer vertex and Eq 6 may be rewritten

$$\begin{array}{c} \sum_{\beta \in N_{\alpha ,\text{in}}}G_{\alpha \beta }(\pi_{\alpha }-\pi_{\beta })+G_{\alpha s}\pi_{\alpha }=G_{\alpha s}\pi_{s}. \end{array}$$ (7)

### 2.3 Continuum approach for the capillary network

Because of its dense and space-filling structure [9], we treat the capillary bed as a porous medium where capillary vessels represent an interconnected network of pores embedded in the cerebral tissue. At the mesoscopic scale, we describe momentum transport using Darcy’s law, which can be obtained from homogenization via volume averaging (see e.g. [22, 23, 37]) of creeping flow at microscopic scale. We have

$$\begin{array}{c} U=-\frac{1}{\mu^{\text{eff}}}K^{\text{eff}}\cdot \nabla P, \end{array}$$ (8)

where **U** is the Darcy velocity (a spatially averaged velocity), *P* is the pressure, *μ*^eff^ is the effective viscosity of blood and $K^{\text{eff}}$ is the permeability tensor. We assume that the capillary network is isotropic and homogeneous, so we have $K^{\text{eff}}=K^{\text{eff}}I$. The effective parameters *K*^eff^ and *μ*^eff^ are calculated by solving microscopic-scale flow (Eqs 6 and 7) at mesoscopic scale [21]. Both coefficients are usually lumped together into a single effective resistivity *K*^eff^/*μ*^eff^ of the network [14, 38]. In this paper, as detailed in Appendix A, the effective permeability *K*^eff^ rather represents the permeability of the network with uniform viscosity, while the effective viscosity *μ*^eff^ represents the impact of the Fåhræus-Lindqvist effect (Eq (3)).

Mass balance in the porous medium is written as

$$\begin{array}{c} \nabla \cdot U=-\sum_{s\in S}\delta (x-x_{s})q_{s}, \end{array}$$ (9)

with *q*_*s*_ the flow rate source term, which results from the coupling condition applied at the coupling point *s* located at the position **x**_*s*_, at any endpoint of feeding arteriolar or draining venular trees (more detail in Section 2.4). *S* represents the set of all sources and *δ*(**x** − **x**_*s*_) the Dirac delta corresponding to the point source *s*.

To obtain a system of equations involving only pressures, we substitute Eq 8 into Eq 9, which yields

$$\begin{array}{c} \frac{K^{\text{eff}}}{\mu^{\text{eff}}}\Delta P=\sum_{s}\delta (x-x_{s})q_{s}. \end{array}$$ (10)

The solution of this partial differential equation is computed using a standard cell-centered finite volume (FV) approach [39, 40] on a Cartesian grid. For convenience, we consider cubic cells of volume *h*^3^, although extension to non-cubic cells is straightforward. For cell *i*, the discretization reads

$$\begin{array}{c} \frac{K^{\text{eff}}h}{\mu^{\text{eff}}}\sum_{j\in N_{i}}(P_{i}-P_{j})=\sum_{s\in S_{i}}q_{s}, \end{array}$$ (11)

where *N*_*i*_ is the set containing the indices of all the neighbours to cell *i*, *P*_*i*_ and *P*_*j*_ are the approximated pressures of cells *i* and *j*, *S*_*i*_ is the set of sources in cell *i* and *q*_*s*_ is the flow rate source term. The quantified pressure *P* defined at the center of a cell is a local averaging of the pressure field. In the visualizations of this paper, we will consider a piecewise constant reconstruction of the pressure in the continuum, although a more precise reconstruction can be obtained by linear interpolation.

### 2.4 Multiscale coupling condition to link arteriolar and venular trees to the capillary bed

So far, we have described two separate frameworks on distinct scales to represent momentum transport in brain microcirculation: a network approach for arteriolar and venular trees and a homogenized continuum representation for the capillary network. Each of these descriptions is self-sufficient when boundary conditions and source terms are fixed for each separately. For instance, if *q*_*s*_ and *π*_*s*_ are known in the network approach, we can assemble the set of linear equations Eqs 6 and 7 into a sparse matrix plus a right-hand side and solve this linear system to obtain the pressure at each inner vertex and thus the flow rate in each vessel. However, some of the boundary conditions are in fact not known, but rather correspond to coupling elements with the continuum description. In this case, a coupling condition must be developed to express the relationship between the pressure *π*_*s*_ at the coupled endpoint of the arteriolar or venular vessel (Eq 7) and the pressures of the local finite volume cells.

Here, we present an approach to couple these two frameworks, which is inspired from that of Peaceman [41], who developed a “well model” for petroleum engineering. In these applications, a wellbore model is coupled with a continuum reservoir description, in which variations at the scale of the underlying structure are not described. The main idea is thus to represent the strong gradients that occur in the vicinity of the wellbore, i.e., within a length scale much smaller than the size of a FV cell, by a singular pressure drop term embedded in the coupling condition. To this end, analytical expressions of the pressure field in the vicinity of the coupling points are used to derive relationships between variables of both frameworks.

**Fig 3:**
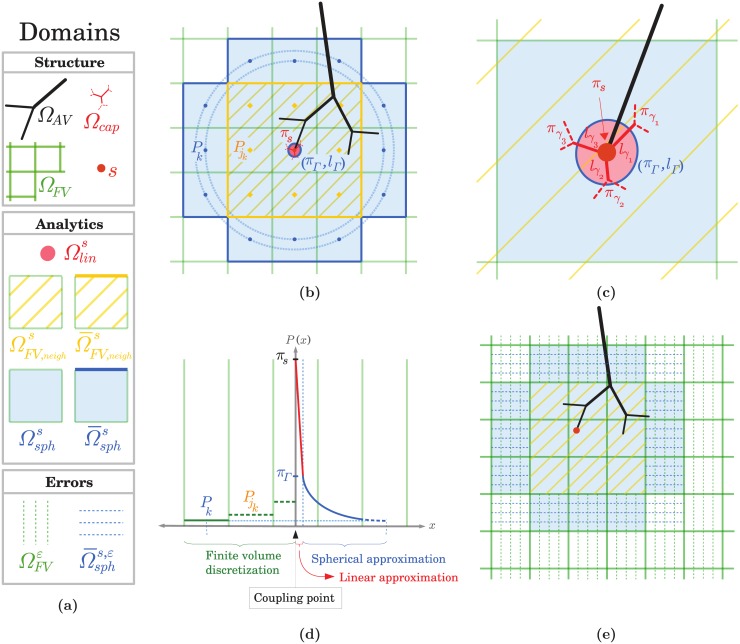
Schematic diagrams of the derivation of the coupling condition between one of the arteriolar or venular endpoints and the continuum representing the capillary bed. **(a)** The legend shows all the domains used for the development of the coupling model with a detailed nomenclature provided in Appendix D. **(b)** An arteriolar or venular tree Ω_*AV*_ (black network) plunges into a FV grid Ω_*FV*_ (green grid). The capillary vessels in Ω_*cap*_ that are connected to the coupling point (highlighted by a red dot) are represented in red. The coupling condition describes the relationship between the network pressure *π*_*s*_ at this coupling point and the pressures of the surrounding cells $\Omega_{FV,neigh}^{s}$, which include the coupled cell. The pressure field is reconstructed in the local neighbourhoods $\Omega_{lin}^{s}$ and $\Omega_{sph}^{s}$ using Eq 20a and 20b. In the most distant cells ${\bar{\Omega }}_{sph}^{s}$, the analytical approximation matches with the FV representation further away. To make visualizations easier, a centered coupling is illustrated. **(c)** Detailed view of the coupled cell. The linear and spherical approximations match at a distance *l*_Γ_ from the coupling point. *π*_Γ_ is the averaged weighted pressure evaluated at this distance. **(d)** 1D pressure profile: FV pressures (green steps) are plotted on the left-hand side while both linear (red) and spherical (blue) approximations are plotted on the right-hand side. **(e)** Domains for the computation of errors. Four regions have been selected to compare hybrid and CN approaches: the arteriolar and venular trees Ω_*AV*_ (black), the coupling points *s* (red), the surrounding cells $\Omega_{sph}^{s,\varepsilon }$ (hatched blue) and the rest of the FV domain $\Omega_{FV}^{\varepsilon }$ (hatched green) which excludes cells where the pressure field must be analytically reconstructed.

In our approach, the pressure field in the neighbourhood of a coupling point is decomposed into two analytical approximations. In the closest region $\Omega_{lin}^{s}$ to the coupling point, a linear variation captures the network structure at the scale of the capillary vessels connected to this point. In $\Omega_{sph}^{s}$, up to two FV cells away from the coupling point, a spherical solution of the continuum equations then described the variation at the scale of *h*, the side of a FV cell. The notations used, the relevant spatial domains and the modelling principles are schematized in Fig 3. A detailed nomenclature of the domains is also provided in Appendix D.

The main advantage of the resulting coupling condition is that it expresses a linear relationship between the pressure at the coupled endpoint of the arteriolar or venular vessel and the pressures of the local finite volume cells. Thus it can be applied to each coupling point independently. Since each coupling is blind to the others, we focus on a single coupling point in this section to explain the development of the coupling model.

Let us consider a single coupling point *s*, at any arteriolar or venular coupled endpoint. The local flow rate *q*_*s*_ corresponds to the coupling term that appears in both Eq 6 for the network approach and Eq 11 for the continuum representation. The coupling condition aims at describing the pressure drop between the pressure *π*_*s*_ at the endpoint of the arteriolar or venular vessel, and the cell pressures ${\left(P_{j}\right)}_{j\in {\bar{\Omega }}_{FV,neigh}^{s}}$ of the FV neighbourhood, where ${\bar{\Omega }}_{FV,neigh}^{s}$ represents the set of cells that surround the coupled one (see Fig 3**(b)**).

We first present an analytical solution of Darcy’s law (Eq 10) close to the coupling point, in the neighbourhood $\Omega_{sph}^{s}$ (Fig 3**(b)**). Assuming a spherical symmetry of the pressure field for a single coupling, we have

$$\begin{array}{c} P(r)=\pi_{ref}-\frac{\mu^{\text{eff}}q_{s}}{4\pi K^{\text{eff}}}(\frac{1}{r}-\frac{1}{l_{ref}}), \end{array}$$ (12)

with *π*_*ref*_ a reference pressure defined at an arbitrary distance *l*_*ref*_ to the source, at microscopic scale, and *r* ≥ *l*_*ref*_ the distance to the coupling point. This solution yields a 1*r*-decrease of the pressure, as displayed in blue on Fig 3**(d)**.

In order to determine *π*_*ref*_ and *l*_*ref*_, a second analytical approximation of the pressure field is introduced for *r* ≤ *l*_*ref*_ at microscopic scale. To this end, we extend the network approach (Eq 6) to the capillaries Ω_*cap*_ connected to the point *s* (see Fig 3**(c)**). Replacing the subscripts *β* that refer to inner vertices of the arteriolar and venular trees, in Eq 6, by subscripts *γ* referring to inner vertices of the capillary bed, this reads

$$\begin{array}{c} \sum_{\gamma \in N_{s}}G_{s\gamma }(\pi_{s}-\pi_{\gamma })=q_{s}. \end{array}$$ (13)

From this equation, we derive a linear pressure drop, as displayed in red on Fig 3**(d)**,

$$\begin{array}{c} \pi_{\Gamma }=\pi_{s}-R_{\Gamma }q_{s}, \end{array}$$ (14)

with the weighted average pressure

$$\begin{array}{c} \pi_{\Gamma }=\frac{1}{\sum_{\gamma \in N_{s}}G_{s\gamma }}\sum_{\gamma \in N_{s}}G_{s\gamma }\pi_{\gamma } \end{array}$$ (15)

and

$$\begin{array}{c} R_{\Gamma }=\frac{1}{\sum_{\gamma \in N_{s}}G_{s\gamma }}. \end{array}$$ (16)

This expression is valid over a length scale *l*_Γ_, which defines the domain $\Omega_{lin}^{s}$ in Fig 3**(c)**. Eq 14 can be rewritten as the pointwise formulation

$$\begin{array}{c} P(r)=\pi_{s}-\frac{\pi_{\Gamma }-\pi_{s}}{l_{\Gamma }}\;r\;\;\text{for}\;r\leq l_{\Gamma }. \end{array}$$ (17)

We then match both analytical approximations by imposing *π*_*ref*_ = *π*_Γ_ in Eq 12. This yields

$$\begin{array}{c} P(r)=\pi_{\Gamma }-\frac{\mu^{\text{eff}}q_{s}}{4\pi K^{\text{eff}}}(\frac{1}{r}-\frac{1}{l_{\Gamma }}), \end{array}$$ (18)

with

$$\begin{array}{c} l_{\Gamma }=\frac{\sum_{\gamma \in N_{s}}G_{s\gamma }}{\sum_{\gamma \in N_{s}}\frac{G_{s\gamma }}{l_{s\gamma }}}. \end{array}$$ (19)

The computation of *l*_Γ_ is detailed in Appendix B.

At this stage, the pressure field is defined at any point of $R^{3}$ in the neighbourhood of the coupling point *s*. Let *r* be the distance from a given point of the neighbourhood to the coupling point *s*. From Eqs 17 and 18, we have

$$P\left(r\right)=\{\begin{array}{lll} \pi_{s}-\frac{\pi_{\Gamma }-\pi_{s}}{l_{\Gamma }}r & \text{for}\;r\leq l_{\Gamma } & \left(20\text{a}\right)\\ \pi_{\Gamma }-\frac{\mu^{\text{eff}}q_{s}}{4\pi K^{\text{eff}}}\left(\frac{1}{r}-\frac{1}{l_{\Gamma }}\right) & \text{for}\;r>l_{\Gamma }, & (20\text{b)} \end{array}$$

as displayed on the right-hand side of Fig 3**(d)**.

The coupling condition is completed by assuming the existence of a domain where both the spherical approximation (Eq 20b and right part of Fig 3**(d)**) and the FV formulation (Eq 11 and left part of Fig 3**(d)**) are valid, so that the pressure of a FV cell of this domain matches with the pointwise spherical expression applied to the distance between the coupling point and the center of the cell (e.g. cell pressure *P*_*k*_ in Fig 3**(d)**). This condition is based on the key assumption that *h* is sufficiently larger than *l*_*cap*_. We further consider that the matching occurs only in the most distant cells of $\Omega_{sph}^{s}$, ${\bar{\Omega }}_{sph}^{s}$. Thus, in any cell *k* in ${\bar{\Omega }}_{sph}^{s}$, the cell pressure is expressed as

$$\begin{array}{c} P_{k}=\pi_{\Gamma }-\frac{\mu^{\text{eff}}q_{s}}{4\pi K^{\text{eff}}}(\frac{1}{d_{k,s}}-\frac{1}{l_{\Gamma }}), \end{array}$$ (21)

where *d*_*k*,*s*_ is the distance between the coupling point and the center of cell *k*.

Combining Eqs 14 and 21, we can obtain an expression of *q*_*s*_ as a function of *π*_*s*_ and ${\left(P_{k}\right)}_{k\in {\bar{\Omega }}_{sph}^{s}}$. To finally close the expression of the coupling condition and express *q*_*s*_ as a function of *π*_*s*_ and ${\left(P_{j}\right)}_{j\in {\bar{\Omega }}_{FV,neigh}^{s}}$, we use the FV formulation (Eq 11) to express the flow conservation at the interface between the cells of the neighbourhood ${\bar{\Omega }}_{FV,neigh}^{s}$ and the rest of the FV domain, i.e. between ${\bar{\Omega }}_{FV,neigh}^{s}$ and ${\bar{\Omega }}_{sph}^{s}$. The FV discretization reads

$$\begin{array}{c} \frac{K^{\text{eff}}h}{\mu^{\text{eff}}}\sum_{k\in {\bar{\Omega }}_{sph}^{s}}(P_{j_{k}}-P_{k})=q_{s}, \end{array}$$ (22)

where $P_{j_{k}}$ represents the pressure of the unique neighbouring cell *j*_*k*_ of *k*, which belongs to ${\bar{\Omega }}_{FV,neigh}^{s}$ (see Fig 3**(b)**).

By combining Eqs 14, 21 and 22, we finally obtain the coupling expression

$$\begin{array}{c} q_{s}=f\left(\pi_{s},{\left(P_{j}\right)}_{j\in {\bar{\Omega }}_{FV,neigh}^{s}}\right), \end{array}$$ (23)

where *f* is the following linear function

$$\begin{array}{c} f\left(\pi_{s},{\left(P_{j}\right)}_{j\in {\bar{\Omega }}_{FV,neigh}^{s}}\right)=\frac{1}{C_{s,\Gamma }}\;\left(\frac{1}{\text{Card}\left({\bar{\Omega }}_{sph}^{s}\right)}\sum_{k\in {\bar{\Omega }}_{sph}^{s}}P_{j_{k}}-\pi_{s}\right), \end{array}$$ (24)

with Card(⋅) representing the cardinal number of a given set and

$$\begin{array}{rr} C_{s,\Gamma }= & R_{\Gamma }-\frac{\mu^{\text{eff}}}{4\pi K^{\text{eff}}}\;(\frac{1}{\text{Card}\left({\bar{\Omega }}_{sph}^{s}\right)}\sum_{k\in {\bar{\Omega }}_{sph}^{s}}\frac{1}{d_{k,s}}\\ & \times -\frac{1}{l_{\Gamma }}-\frac{4\pi }{h\times \text{Card}\left({\bar{\Omega }}_{sph}^{s}\right)}). \end{array}$$ (25)

Recall that the FV cells are assumed to be cubic here, although the extension to parallelepipedic cells is straightforward.

The development above is valid for both centered and off-centered couplings, as in Fig 3**(e)**. However, the FV scheme (Eq 11) is not sensitive to the position of the coupling point in a single source cell. Thus, the resulting FV pressure field is independent of the source position in a coupled cell. To properly take into account the off-centering of a coupling point *s* in the FV part of the hybrid approach, the coupling term *q*_*s*_ is redistributed on each cell *i* of the neighbourhood $\Omega_{FV,neigh}^{s}$ with new partial source terms defined by

$$\begin{array}{c} q_{s,i}=\tau_{i,s}q_{s}, \end{array}$$ (26)

according to a partition coefficient *τ*_*i*,*s*_

$$\begin{array}{c} \tau_{i,s}=\frac{v_{i}}{h^{3}}, \end{array}$$ (27)

where *v*_*i*_ is the volume of the intersection between the cell *i* and a fictitious mesh cell centered on the coupling point (see S1 Fig in Supporting Information). For a given source *s*, this definition implies that ∑*_i_* *τ_i,s_* = 1, yielding ∑*_i_* *q_i,s_* = *q_s_*, and that, among the 27 cells of $\Omega_{FV,neigh}^{s}$ no more than 8 verify *τ_i,s_* ≠ 0, and *q_i,s_* ≠ 0. From the point of view of the FV scheme (Eq 11), a given cell i of the FV domain is impacted by any coupling located in its neighbourhood ${\bar{N}}_{i}$, which includes the cells sharing a single corner with *i*. Thus, Eq 11 is can be rewritten as

$$\frac{K^{\text{eff}}h}{\mu^{\text{eff}}}\sum_{j\in N_{i}}\left(P_{i}-P_{j}\right)=\sum_{s\in S\left({\bar{N}}_{i}\right)}q_{s,i},$$ (28)

where $S\left({\bar{N}}_{i}\right)$ is the set of coupling points in ${\bar{N}}_{i}$. Combining Eqs 24 and 26–28, Eq 28 finally reads

$$\begin{array}{ll} \frac{K^{\text{eff}}h}{\mu^{\text{eff}}}\sum_{j\in N_{i}}\left(P_{i}-P_{j}\right) & =\sum_{s\in S\left({\bar{N}}_{i}\right)}q_{s,i}\\ & =\sum_{s\in S\left({\bar{N}}_{i}\right)}\tau_{i,s}q_{s}\\ & =\sum_{s\in S\left({\bar{N}}_{i}\right)}\tau_{i,s}f\left(\pi_{s},{\left(P_{j_{s}}\right)}_{j_{s}\in {\bar{\Omega }}_{FV,neigh}^{s}}\right). \end{array}$$ (29)

### 2.5 Matrix structure and numerical implementation

Due to the linearity of the hybrid model, we can compute the flow in the whole domain by solving a single linear system, assembled from the equations describing the FV (Eq 28), the network (Eqs 6 and 7) and the coupling (Eqs 23–25). This yields a sparse matrix which is composed of seven parts: two symmetric sparse blocks for the description of the FV and the network, of size (number of cells)^2^ and (number of inner vertices)^2^, respectively; a diagonal block, of size (number of couplings)^2^; and four supplementary off-diagonal blocks for the description of the relationships between the couplings points and both the network and the continuum (see more detail on the matrix structure in Appendix C). The right-hand side describes the boundary conditions imposed at the inlets and outlets of the arteriolar and venular trees, and at the six faces of the FV domain.

The sparsity of the linear system allows us to take advantage of the efficient and scalable numerical tools in the PETSc library [42]. We use a block-Jacobi preconditioner, which is very well-suited to parallel computations, and the system is solved via the bi-conjugate gradient iterative method. The code was developed from scratch in C++ and is fully parallelized using MPI, PETSc [43] and the ParMETIS library for parallel graph partitioning of the network [44]. The code is designed for High Performance Computing (HPC) and runs on EOS supercomputer (CALMIP, France). Regular automatic testing, using CxxTest (http://cxxtest.com/), ensures reliability of the code [45].

Alternately, this code can be used to run computations where all the vessels, including the capillaries, are modelled by the network approach. These complete network (CN) simulations will be used in Section 3 to generate reference fields enabling the evaluation of the accuracy of the hybrid approach.

### 2.6 Architecture of capillary networks and modelling configurations

**Fig 4:**
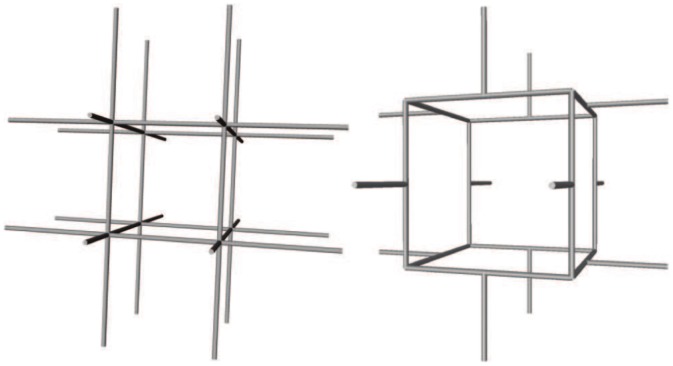
Visualization of the model three-dimensional lattices for the simplified representation of the capillary networks. The elementary patterns of 6- and 3-regular networks are presented on the left- and right-hand sides, respectively. Each inner vertex of a 6-regular network has 6 neighbours, while an inner vertex of a 3-regular network has always 3 neighbours. These patterns can be duplicated as necessary in each direction, depending on the desired size of the capillary network.

With a view to model validation, calculations are performed here for two types of model capillary networks, whose names were chosen according to graph theory [46]: (1) 6-regular networks, i.e., where each vertex is connected to 6 neighbours; and (2) 3-regular networks, which comply to the physiological connectivity in healthy capillary networks (see Fig 4). The use of synthetic capillary networks enables both to solve the problem of anatomical data availability at this scale and to verify all the modelling assumptions made in the present paper (isotropy and uniform permeability). Furthermore, using anatomical capillary datasets would generate uncertainties in the computation of the effective parameters that characterize the capillary bed in the continuum representation. This would add errors in the hybrid resolution that would be indiscernible from errors induced by the continuous/discrete coupling model, going against our wish to investigate the intrinsic impact of the multiscale coupling model on large-scale flow fields.

In both 6- and 3-regular networks, the capillaries have uniform lengths, *l_cap_* = 50 *μ*m in 6-regular networks and *l_cap_* = 30.62 *μ*m in 3-regular networks, so that the two network types have similar vascular densities. Capillary diameters are either uniform (*d_cap_* = 8 *μ*m) or distributed following a Gaussian distribution with a mean diameter of 5.91 *μ*m and a standard deviation of 1.30 *μ*m, which is characteristic of human capillary networks [8]. The corresponding effective parameters *K*^eff^ and *μ*^eff^ for the continuum representation are presented in Table 1 (more detail in Appendix A).

**Table 1:** Effective parameters for the continuum representation of the model capillary networks. This table summarizes the values of effective permeability *K*^eff^ and viscosity *μ*^eff^ used in this paper (Section 2.6). Details about the computation of these coefficients are provided in Appendix A.

		*d_cap_* = 8 *μ*m	*d*_*cap*_∼ Gaussian distr.
6-regular network	*l_cap_* = 50 *μ*m	*K*^eff^ = 4.02 × 10^−14^ m^2^	*K*^eff^ = 1.21 × 10^−14^ m^2^
		*μ*^eff^ = 5.15 × 10^−3^ Pa · s	*μ*^eff^ = 5.88 × 10^−3^ Pa · s
3-regular network	*l_cap_* = 50 *μ*m	*K*^eff^ = 2.56 × 10^−14^ m^2^	*K*^eff^ = 6.72 × 10^−15^ m^2^
		*μ*^eff^ = 5.15 × 10^−3^ Pa · s	*μ*^eff^ = 6.05 × 10^−3^ Pa · s
	*l_cap_* = 30.62 *μ*m	*K*^eff^ = 6.61 × 10^−15^ m^2^	*K*^eff^ = 1.58 × 10^−14^ m^2^
		*μ*^eff^ = 5.15 × 10^−3^ Pa · s	*μ*^eff^ = 6.14 × 10^−3^ Pa · s

**Fig 5:**
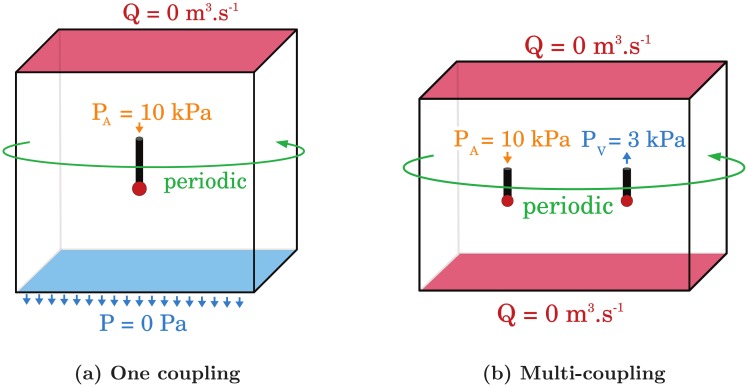
Schematics of the boundary conditions imposed for the validation tests. **(a) and (b)** The black boxes represent either the capillary bed in CN configuration or the continuum in the hybrid approach. For comparison, the boundary conditions imposed at the boundaries of the capillary bed in the CN simulations are similar to those imposed at the faces of the continuum in the hybrid approach. The black cylinders represent the arteriolar and/or venular vessels. The in- and outflow are illustrated by arrows. The arteriolar and venular endpoints highlighted by red dots are the coupling sites, which are connected to capillaries in CN configuration or connected to the continuum using the coupling model in the hybrid approach. **(a)** The boundary conditions are schematized for the simplest configuration where a single arteriolar vessel is coupled with a cubic capillary bed/continuum. A pressure drop of 10 000 Pa is imposed between the arteriolar inlet and the bottom face of the capillary bed/continuum. A condition of impermeability (i.e. no flow) is imposed at the top face, and periodic conditions are imposed in the two other directions (left-right and front-back). **(b)** The boundary conditions are schematized for a multi-coupling configuration, where one arteriolar and one venular vessels are coupled with a parallelepipedic capillary bed/continuum. A pressure drop of 7 000 Pa is imposed between the arteriolar inlet and the venular outlet. A condition of impermeability (i.e. no flow) is imposed at the top and bottom faces, and periodic conditions are imposed in the two other directions (left-right and front-back).

We will first consider basic configurations that involve less than two couplings. For a single coupling, an arteriole penetrates into a cubic 6-regular capillary bed of size (2.25 mm)^3^ containing about 284000 vessels. For the case of two coupling endpoints, an arteriole and a venule plunge into a parallelepipedic capillary bed of size 5.5 mm × 2.25 mm × 2.25 mm containing 557000 vessels. Because we want to investigate the impact of the cell size *h* on the accuracy of the model, we choose to ensure mesh convergence by considering large capillary domains. Thus we also avoid the potential interference of boundaries with the reconstruction of the pressure and flow rate fields. The arteriolar and venular vessels are *l_AV_* = 200 *μ*m long and *d_AV_* = 15 *μ*m in diameter. The boundary conditions are illustrated in Fig 5. For consistency, the boundary conditions imposed at the boundaries of the capillary bed in the CN simulations are similar to those imposed at the faces of the continuum in the hybrid approach. In the case with two couplings (Fig 5**(b)**), we impose a constant pressure drop *δ*P = 7000 Pa between the arteriolar inlet and the venular outlet [15, 47]; impermeability (zero flow) on the top and bottom of the capillary network; and periodic conditions in the two other directions. The condition of impermeability imposed at the top of the domain is physiologically relevant to describe the surface of the cortex. The same condition is usually imposed at the bottom, e.g. [14, 15]. For a single coupling (Fig 5**(a)**), boundary conditions are similar with a zero pressure condition at the bottom of the capillary bed, inducing a global pressure drop *δP* = 10000 Pa.

Second, we will consider a more realistic case, which involves the anatomical arteriolar and venular trees presented in Fig 2**(a)**. This dataset has been previously obtained by Cassot *et* *al*. [8] from sections of a human brain, from the Duvernoy collection [7]. The dataset supporting this article is provided in a supplementary data file. It is composed of a venular tree in between two arteriolar trees, containing a total of 563 vessels and displaying 274 coupling points with a realistic spatial distribution, as displayed on Fig 2**(b)**. Arteriolar and venular vessel diameters are in the range *d_AV_* ∈ [7.5 *μ*m; 42.6 *μ*m]. They are connected to a 3-regular capillary network of size 4 mm × 3 mm × 1.5 mm containing 1.6 million vessels. The boundary conditions are identical to the ones defined above for two couplings, as shown in Fig 5**(b)**, except that the pressure drop of 7 000 Pa is imposed between the two inlets of the arteriolar trees and the outlet of the venular tree.

### 2.7 Error metrics

To evaluate the accuracy of the model, we compare results of the hybrid approach with CN simulations, where blood flow in all vessels is described by the network approach (Eqs 6 and 7). We consider the four domains of interest illustrated in Fig 3**(e)**: the source points *s*, the whole arteriolar and venular trees Ω_*AV*_, the distant FV neighbourhoods $\Omega_{sph}^{s,\varepsilon }$ of each coupling point *s* and the rest of the continuum $\Omega_{FV}^{\varepsilon }$. In the arteriolar and venular trees, variables are identical in both approaches, therefore facilitating comparison. To compute errors in subdomains of the continuum, the capillary pressures in the CN approach are averaged over volumes corresponding to the FV cells in the hybrid approach, and capillary flow rates are averaged over surfaces which correspond to the interfaces between two discretization cells.

Let Ω be one of the four domains of interest mentioned above. Ω contains two sets of components: the set Ω^*p*^ of network vertices or FV cells, where pressures are defined, and the set Ω^*q*^ of network vessels or interfaces between two FV cells, where flow rates are defined. For all component *ω* of Ω^*p*^, the local pressure error $\varepsilon_{\omega }^{p}$ reads

$$\begin{array}{c} \varepsilon_{\omega }^{p}=\frac{\left|p_{\omega ,\text{H}}-p_{\omega ,\text{CN}}\right|}{p_{\omega ,\text{ref}}}, \end{array}$$ (30)

where *p* represents the pressure value, the subscripts H and CN refer to the hybrid and CN approaches, and the subscript ref refers to the reference value used for normalization. For all component *ω* of Ω^*q*^, the local flow rate error reads

$$\begin{array}{c} \varepsilon_{\omega }^{q}=\frac{\left|q_{\omega ,\text{H}}-q_{\omega ,\text{CN}}\right|}{q_{\omega ,\text{ref}}}, \end{array}$$ (31)

where *q* represents the flow rate value. The global errors characterizing Ω are further defined as

$$\varepsilon_{\Omega }^{p}=\frac{\sum_{\omega \in \Omega^{p}}\varepsilon_{\omega }^{p}}{\text{Card}(\Omega^{p})}$$ (32)

and

$$\varepsilon_{\Omega }^{q}=\frac{\sum_{\omega \in \Omega^{q}}\varepsilon_{\omega }^{q}}{\text{Card}(\Omega^{q})}.$$ (33)

For the study of errors distribution, we use the local errors in Eqs 30 and 31. To avoid underestimation, these errors are normalized by local reference values, so that *p*_*ω*,ref_ = *p*_*ω*,CN_ and *q*_*ω*,ref_ = *q*_*ω*,CN_ for all *ω*. For overall error estimation, we use the global errors of Eqs 32 and 33. In this case, errors are normalized by the global pressure drop *δP* across the sample considered or the global flow rate *q*_*A*_ at the inlet of the arteriolar vessel, so that *p*_*ω*,ref_ = *δP* and *q*_*ω*,ref_ = *q*_*A*_ for all *ω* in Eqs 30 and 31. These global errors are particularly well-suited to compare the variation of errors among several test cases.

## 3 Results and discussion

### 3.1 Validation of the hybrid approach for basic configurations

**Fig 6:**
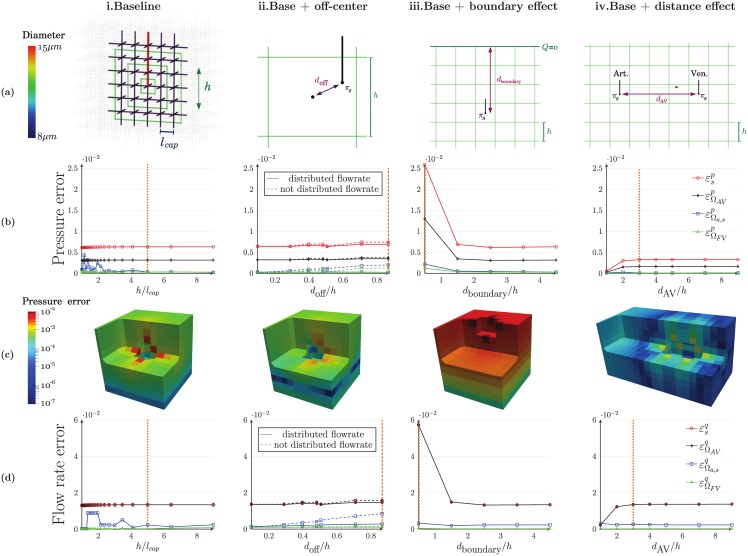
Comparison of hybrid and reference CN approaches for simple configurations. In the CN version, the arteriolar and venular trees are connected to a 6-regular capillary network. In the hybrid approach, they plunge into an equivalent porous medium which is discretized in FV cells of size *h*. **(a)** *Schematics of the basic tests*: in the baseline configuration **i**, a single coupling is imposed at the center of the coupled cell. In the following configurations (**ii** to **iv**), we independently study the robustness of the model by imposing three geometrical constraints that typically occur in brain microcirculation: off-centering of the coupling in a cell (**ii**), effect of domain boundaries (**iii**), and effect of the distance to a second coupling point (**iv**). These effects are characterized by three distances from the coupling point to: the center of the coupled cell (**ii**, distance *d*_off_), the upper domain boundary (**iii**, distance *d*_boundary_), and another coupling point (**iv**, distance *d*_*AV*_). **(b)** *Global pressure errors*: for each configuration, global pressure errors are computed in the four domains of interest defined in Fig 3**(e)**. The errors are normalized by the global pressure drop *δP*. **(c)** *Local pressure errors*: the distributions of the local pressure errors are displayed for the ratios *hl*_*cap*_ indicated by orange dotted lines in **(c)**. **(d)** *Global flow rate errors*: the flow rate errors are computed in the same four domains of interest. The errors are normalized by the global incoming flow rate *q*_*A*_.

Here, our goal is to assess the robustness of the coupling model and the relevance of strategies that have been adopted during its development. The first test case analyzes the impact of the size of FV cells on the accuracy of the model (Fig 6**i(a)**). Three other configurations test different kinds of perturbations that typically occur in brain microcirculation: off-centering (Fig 6**ii(a)**), effects of boundary conditions (Fig 6**iii(a)**) and coupling points coming close together (Fig 6**iv(a)**). The accuracy of the model is determined by comparing the hybrid and CN approaches, using the global error metrics, Eqs 32 and 33, for the four domains of interest illustrated in Fig 3**(e)**. While these errors may not be as representative as local errors, this choice enables us to compare the different graphs displayed in Fig 6 and to conclude about the importance of the impact of each perturbation.

An important hypothesis of the coupling model is that the size *h* of a mesh cell is sufficiently larger than the characteristic length *l*_*cap*_ of the capillaries—so that Eq 21 is valid. The most sensitive area to this condition is *a priori* the distant neighbourhood $\Omega_{sph}^{s,\varepsilon }$ (Fig 3**(e)**), which corresponds to the intermediate zone between the analytical approximations and the FV formulation. In the first test case (Fig 6**i**), we focus on identifying the threshold value of *h*/*l*_*cap*_ for which the FV cells are large enough. As expected, the errors $\varepsilon_{\Omega_{sph}^{s}}^{p}$ and $\varepsilon_{\Omega_{sph}^{s}}^{q}$ fluctuate for small values of *h*/*l*_*cap*_ in the coupling neighbourhood, then decrease with a stabilization for *h*/*l*_*cap*_ close to 4 or h ∼ 200 *μ* m (Fig 6**i(b)** and 6**i(d)**). For comparison, the pressure and flow rate errors have also been computed when imposing a simple condition of pressure continuity at the interface between the arteriolar vessel and the continuum (see S2 Fig in Supporting Information). In this case, all errors significantly increase with the ratio *h*/*l_cap_*. In particular, the pressure and flow rate errors at the coupling point are ~ 29% and ~ 150%, respectively, for *h*/*l_cap_* = 4. They continue to increase for larger values of *h*/*l_cap_*, reaching ~ 45% and ~ 250%, respectively, for *h*/*l_cap_* = 9. This behaviour highlights the difference in the physical meanings between the pressure of the coupled FV cell and the pressure at the endpoint of the arteriolar vessel, which is critical for large values of *h*/*l_cap_*. By contrast, the multiscale coupling model included in our approach takes into account the upscaling transition between the arteriolar vessel and the continuum. Moreover, other geometrical perturbations such as non-uniform capillary diameters distribution, a non-vascular zone around the arteriolar vessel representing the Virshow-Robin space [7] or a different architecture of the capillary bed have no influence on the threshold value of *h*/*l_cap_* (S3 Fig in Supporting Information). In the remainder of this paper, we therefore always use *h*/*l_cap_* ≥ 4.

**Fig 7:**
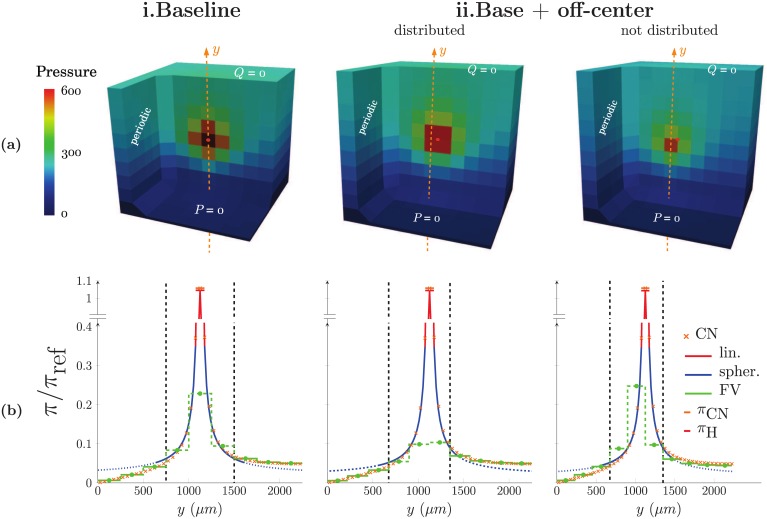
Comparison of pressure fields computed via hybrid and CN approaches. **(a)** The pressure fields are displayed for two configurations of interest: **(i)** centered and **(ii)** off-centered single coupling of an arteriolar vessel in a 6-regular capillary network. The coupling point is indicated by a large orange dot. In the off-centered case, the coupling condition is either distributed among neighbouring cells $\Omega_{FV,neigh}^{s}$ or not. **(b)** Pressure profiles are plotted along a line of interest, which is indicated by orange dashes in **(a)**. The reference pressure field from CN approach is represented by orange crosses. Green steps represent cell pressures of the FV representation, blue and red lines respectively represent linear and spherical approximations. Black dashed lines represent the limit of $\Omega_{FV,neigh}^{s}$, where pressure field is approximated by analytical solutions. The reconstructed pressure field of the hybrid approach is finally obtained by combining solid lines. The asymmetry of the field far away from the source is caused by the imposed boundary conditions at the limits of the domain. Here *π*_ref_ = 5 000 Pa is a reference pressure value used for nondimensionalization.

As displayed in Fig 6**i(c)**), for a ratio *h*/*l*_*cap*_ = 5, i.e. *h* = 250 *μ*m, the pressure errors in the capillary medium $\Omega_{\Omega_{FV}}^{\varepsilon }$ are lower than 1% everywhere when using the multiscale coupling model, showing its accuracy. Furthermore, the reconstruction of the pressure field using the analytical approximations (Eqs 14 and 12) in the vicinity $\Omega_{FV,neigh}^{s}$ of the coupling point is in very good agreement with the reference CN pressure (Fig 7**i**). As expected, the FV representation is accurate beyond two cells away from the coupling but does not describe the strong gradients in $\Omega_{FV,neigh}^{s}$, underestimating the pressure in the coupled cell. The coupling model correctly captures these gradients since the pressures *π*_*CN*_ and *π*_H_ at the coupling point are in very good agreement, with a global error of 0.7%. By comparison, the difference between these two values is 100 times larger when using a simple condition of pressure continuity, with a global error of 33% (S2 and S4 Figs in Supporting Information). Moreover, because the additional pressure drop building up around the coupling point is not accounted for, the pressure at the arteriolar endpoint is highly underestimated (*π*/*π*_ref_ = 0.39 vs. 1.05 in the CN approach, see S4 Fig). Thus, this local singular pressure drop significantly contributes to the global resistance of the capillary bed, which has been recently shown to dominate the overall intracortical resistance in both human and mice [31, 48]. In other words, this microscopic scale effect, intrinsically linked to the transition between the tree-like arterioles and the mesh-like capillaries, has a huge impact on the pressure field at macroscopic scale.

We now ask whether the off-centering of the arteriolar or venular endpoints in the finite-volume cell affects the accuracy of the result. Off-centering of coupling points can indeed induce asymmetries of the pressure field with regard to the FV grid. To deal with this, we introduce partial sources (Eq 28) to distribute the source term among the cells affected by the well model and compare the results with computations where the flow rate is not distributed. In Fig 6**ii**, we analyze the impact on the pressure and flow rate errors (Fig 6**ii(b)** and 6**ii(d)**) by progressively increasing the distance *d*_off_ to the FV cell center in the range dofflcap∈[0.09,32]. The ratios *d*_off_/*l*_*cap*_ = 0.5, dofflcap=22 and dofflcap=32 correspond to extrema of the off-centering with couplings occurring at the intersection between 2, 4 and 8 cells. To make such intersections possible, the continuum is discretized in 10 × 10 × 10 cells, i.e. *h* = 225 *μ*m. In this way, the corners of the FV cells in the hybrid approach coincide with bifurcations of the 6-regular underlying capillary bed in the CN configuration. We see that ignoring the distribution of *q*_*s*_ results in an increase of errors, with errors twice as large in the FV neighbourhood ($\varepsilon_{\Omega_{sph}^{s}}^{p}$ and $\varepsilon_{\Omega_{sph}^{s}}^{q}$, plain versus dotted lines in Fig 6**ii**). By reconstructing the analytical approximation of the pressure field in the vicinity of the coupling (Fig 7**ii**), we see that the coupling model without distribution of *q*_*s*_ cannot capture the asymmetry of the pressure field. This induces slight over- and underestimations on each side of the coupling point that persist even far away from the source. On the contrary, the reconstructions with *q*_*s*_ distributed are in very good agreement with the reference CN computations.

Next, we assess the influence of boundary conditions in Fig 6**iii**. The coupling point is initially positioned at the center of the domain (*d*_boundary_/*h* = 4.5) and progressively brought close to a boundary (*d*_boundary_/*h* = 0.5) where impermeability conditions have been imposed. Similarly to the first case, *h* = 250 *μ*m here for the purpose of working with centered couplings and avoid potential errors induced by off-centering. Both pressure and flow rate errors (Fig 6**iii(b)** and 6**iii(d)**) show that our model is robust provided that at least one cell separates the coupling point from the domain boundary, ensuring that the neighbourhood $\Omega_{FV,neigh}^{s}$ of the coupling point does not intersect it. While the model is less accurate when the coupling occurs in the boundary cell, the increase in errors is small (up to 2.5% in pressure and 6% in flow rate).

Finally, we study in Fig 6**iv** the impact of the distance between an arteriolar coupling and a venular coupling on results, as the density of coupling points can be locally very large in the human cortex (Fig 2). Initially separated by 9 cells of side *h* = 250 *μ*m (*d*_AV_/*h* = 9), the two couplings are brought closer together until they are in neighbouring cells (*d*_AV_/*h* = 1). In Fig 6**iv(b)** and 6**iv(d)**, both pressure and flow rate errors show that the hybrid approach is in very good agreement with the CN reference solution, even for short distances that break the spherical symmetry of the pressure field. For the ratio *d*_AV_/*h* = 1, the strong decrease of errors can be explained by the fact that each coupled cell belongs to the neighbourhood $\Omega_{FV,neigh}^{s}$ of the other one. In this case, a shunt appears at the scale of the FV cells and the pressure field is uniform outside the coupling region.

**Fig 8:**
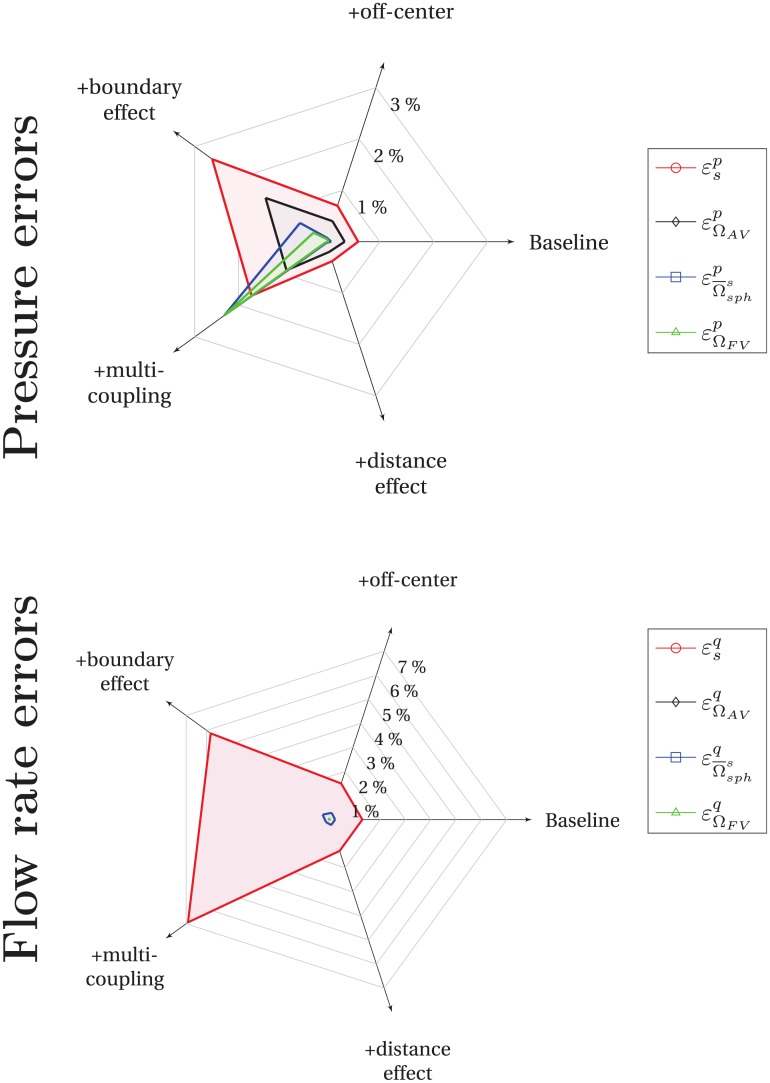
Summary of errors computed for simple configurations, for *h*/*l_cap_* > 4. In Section 3.1, pressure and flow rate errors have been computed for five simple configurations (Baseline, Base+off-center, etc). For *h*/*l_cap_* > 4, maximal errors are indicated by orange dashed lines in each individual graph of Fig 6 and S5 Fig, and combined here in the same diagram.

In summary, the hybrid approach requires a coupling model much more complex than a simple condition of pressure continuity to capture the strong gradients around coupling points. The coupling condition presented in this paper fills this gap since it is robust to the geometrical perturbations that typically occur in the brain microcirculation. For more than two coupling points, we also verified that the model is accurate in similar idealized configurations for up to 10 couplings occurring in the same cell (S5 Fig in Supporting Information). For comparison, the results are summarized in Fig 8. The values reported correspond to the maximal errors computed for *h*/*l_cap_* > 4 (orange dashed lines displayed on each previous graphs—Fig 6 and S5 Fig). With regard to pressure errors, the hybrid approach always shows very good agreement with the CN approach, with no error larger than 3%. The largest errors are observed for the configuration involving multiple couplings in one FV cell, suggesting that the accuracy of the reconstruction of the pressure field in the continuum is limited by the density of couplings per cell. This trend is confirmed when considering flow rate errors in Fig 8. In the next section, we explore this in more detail through a more realistic case representative of the architecture of the human vascular network, where up to 5 couplings occur in the same cell.

### 3.2 Application to larger realistic networks involving anatomical arteriolar and venular trees and synthetic capillary networks

**Fig 9:**
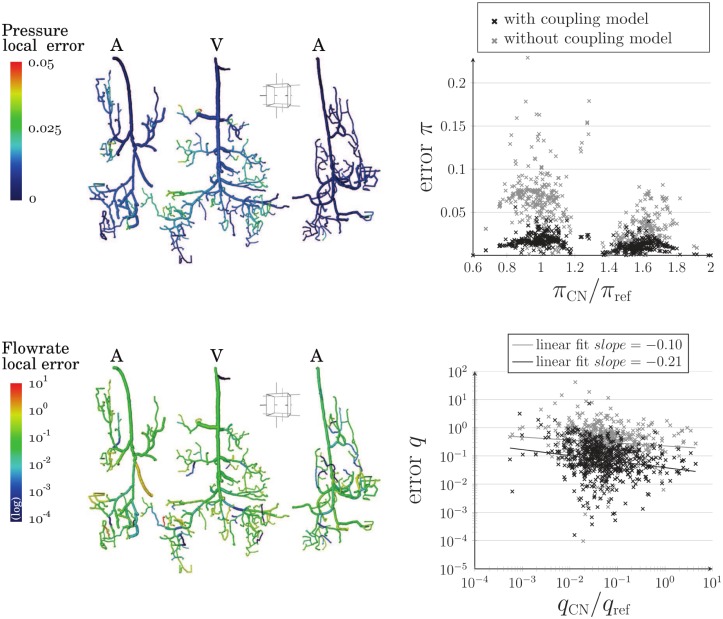
Distribution of pressure and flow rate local errors by comparing the hybrid and CN approaches in the large realistic configuration. **(a)** Pressure and flow rate error maps in arteriolar and venular trees are presented. **(b)** In black crosses, local errors are plotted against local pressure or flow rate reference values. Additional errors are represented as gray crosses corresponding to computations without the proposed coupling model (pressure continuity at coupling sites). Here, *π*_ref_ = 5 000 Pa and *q*_ref_ = 5 × 10^−12^ m^3^ · s^−1^ are reference pressure and flow rate values used for nondimensionalization.

The errors presented in Fig 9 refers to a more realistic configuration involving the three anatomically accurate arteriolar and venular trees displayed in Fig 2**(a)**, which plunge into a 3-regular capillary network with Gaussian diameters distribution and uniform length *l_cap_* = 30.62 *μ*m. The corresponding continuum is discretized in FV cells of side *h* = 122.5 *μ*m, i.e. *h*/*l*_*cap*_ = 4, ensuring the validity of the coupling model. In this case, the global flow rate error, Eq 33, computed at coupling points is very low (<0.26%). Consequently, this also ensures a very good accuracy in the continuum. To go further, we analyze the distribution of local errors, Eqs 30 and 31, in the arteriolar and venular trees.

The pressure error field (Fig 9**(a)**) shows that the hybrid approach remains accurate, with no error greater than 6%. Flow rate is much more sensitive, with errors ranging from 0.001% to 100%. These large values can be explained by the fact that pressure and flow rate fields are highly correlated (Eq 1) in the sense that slight pressure perturbations at the endpoints of a given vessel can induce very high flow rate error, especially for low pressure drops (low flow rates). In spite of this, considering both pressure and flow rate errors in Fig 9**(b)**, we clearly observe the benefit of using the coupling model. This is confirmed when considering absolute values instead of relative errors (S6 Fig in Supporting Information).

**Fig 10:**
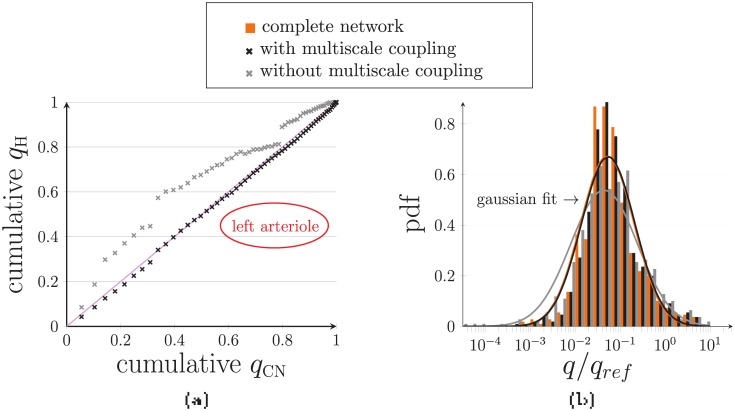
Comparison between the hybrid and CN approaches in the large realistic configuration. **(a)** The cumulative flow rates are compared for both hybrid and CN approaches in the left arteriole. The same conventions as in Fig 9 are adopted. **(b)** The probability density functions are presented for flow rate using the hybrid approach with (black) or without (gray) the coupling model, and CN approach (orange). Here, *π*_ref_ = 5 000 Pa and *q*_ref_ = 5 × 10^−12^ m^3^ · s^−1^ are reference pressure and flow rate values used for nondimensionalization.

Moreover, the large errors highlighted in vessels with low flow rates have a minor impact on the spatial distribution of the source flow rates. This can be verified by comparing the cumulative flow rates, e.g. in the left arteriole (Fig 10**(a)**), deduced from the hybrid approach and CN computations. For that purpose, the source flow rates *q*_*s*_ are first sorted in ascending order and the set of cumulated flow rates is defined for *n* in {1, …, Card(*S*)}, where *S* is the set of sources, by

$$\begin{array}{c} Q_{s}^{n}=\sum_{s=1}^{n}q_{s}. \end{array}$$ (34)

This means that $Q_{s}^{\text{Card}(S)}$ must be equal to the flow rate *q*_*A*,*l*_ injected at the entry of the left arteriole. Finally, the results are normalized by *q*_*A*,*l*_. They show very good agreement with CN computations, which is not the case when considering a simple condition of pressure continuity in the hybrid approach (Fig 10**(a)**). Moreover, probability density functions of the flow rate in the network vessels (Fig 10**(b)**) also show very good accuracy as the Gaussian fits overlap, contrary to those obtained without coupling. These results are particularly interesting because the standard complete network approach has been validated only in a statistical sense, rather than a vessel-to-vessel basis, by comparison with *in vivo* data [20].

Finally, the results presented in this section confirm that the coupling model is very accurate, even for multiple couplings.

### 3.3 Computational performance

In order to estimate the performance gain of the parallel design of the code, blood flow was computed in a network containing more than 10 millions of vertices. This lattice was obtained by periodically duplicating the pattern of three arteriolar and venular trees (Fig 9). The final network contains 10.1 million of vertices in the CN version: 50000 arteriolar and venular vertices and 10.05 million capillary vertices, these latter being homogenized into 51000 mesh cells in the hybrid approach. The computation was run on 20 cores *Intel(R) Xeon(R) CPU E5-2680 v2 @ 2.80GHz* of the EOS supercomputer (CALMIP, France). While the CN approach takes about 6 min, the hybrid approach takes only 1 s, implying a ×360 acceleration. This important gain in computation time is explained by two facts: (1) the number of unknowns is reduced by a factor of ∼200 in the hybrid approach; (2) the unstructured architecture of the capillary network is replaced by a structured grid, which requires much less numerical treatment. By using a simple linear projection of this result, the flow computation in the whole human cortex (∼1000 cm^3^ [49] with ∼10 000 vessels per mm^3^ [8], i.e. 10 billions of vessels) would take about four days with the CN approach while it would take ∼15 minutes with the hybrid approach. This demonstrates that the combination of a HPC code with the hybrid approach is a powerful tool that pushes the current limits of computational performance for simulating blood flow in the brain microcirculation.

### 3.4 Comparison with previous work

Now that we have established the relevance and the numerical strength of our approach, in this section we emphasize the main methodological differences between our model and previous work.

A few authors have introduced hybrid approaches where the arteriolar and venular trees are represented by a discrete model and the capillary network as a continuum [27]. However, the need for a proper coupling condition at the interface between the two frameworks has never been discussed, even in the context of cardiac microcirculation where multiscale computational approaches have been used for a longer time [25, 26, 38]. Reichold *et al.* [14] got around this difficulty by replacing the capillary mesh by a coarser network with identical resistivity. The advantage of such method is to decrease the number of degrees of freedom of the problem, still using a complete network approach. However, this coarsening results in a larger capillary length. Thus the 1*r* pressure relaxation highlighted in this paper around the couplings is likely shifted to larger length scales and the strong pressure gradients around the couplings at microscopic scale may not be properly captured.

One simple alternative idea to capture these strong gradients would be to sufficiently refine the finite volume mesh close to the coupling points. However, the solution of the diffusion equation in the FV scheme (Eq 8) would impose a 1*r*-decrease of the pressure in the vicinity of the coupling point, overlooking the linear variation at microscopic scale, below *l*_*cap*_. Furthermore, even if this were physically accurate, couplings are relatively dense (Fig 2**(a)**), implying that such a refinement would be computationally limiting. This might still be tractable on a larger number of cores, but only to calculate blood flow in moderately large portions of the brain, without the possibility to further consider mass transport modelling or nonlinear effects in the blood rheology.

In the present hybrid approach, we used analytical solutions close to the sources to derive a proper coupling model. This idea was introduced by Peaceman in the context of petroleum engineering [41], in order to couple wellbores with two-dimensional finite volume models representing the flow in an oil reservoir. It was later extended to deal with more complex situations, e.g. anisotropic continuum, non-Darcy flow, three-dimensional models, finite element methods [50–52]. However, because of differences in scales between oil reservoirs and the human cortex (see S7 Fig in Supporting Information), important modifications to the original well models were made. First, the sources and sinks terms are lineic in Peaceman’s approach, while our conceptual model involves point terms. Thus analytical solutions in classical well models exhibit cylindrical symmetry, while the symmetry is spherical here. More importantly, the reference radius and wellbore pressure used for the analytical solution in Peaceman’s model [41] are straightforward because the well diameter is much larger than the pores [53]. In microvascular networks, the diameter of a coupled arteriole or venule is ~ 5 times smaller than the characteristic length of a capillary vessel [9]. Consequently, our model required an additional analytical approximation of the pressure drop in the direct vicinity of couplings, Eq 14, to capture microscopic effects. In addition, the FV cells in an oil reservoir are a thousand times larger than the well diameter [53], while they are only ten times larger than the characteristic length of a capillary in the present work. We therefore expanded the coupling condition to a larger neighbourhood $\Omega_{FV,neigh}^{s}$ around coupling points. In doing so, the model yields an additional local linear system per coupling, which involves the coupled cell and its 26 neighbours. Even if this strategy is constraining, it is a reliable way of improving the accuracy of the model [54], which is similar to extending the stencil in computational methods. Moreover, it enables us to deal with off-centered couplings by distributing the coupling condition among these particular cells, Eqs 27 and 28 (see similar strategies in [55]).

## 4 Conclusions and perspectives

In this paper, we presented a hybrid multiscale approach to simulate blood flow in large volumes of the brain microcirculation. This approach is hybrid in the sense that it couples a network representation of the arteriolar and venular trees to a homogenized continuum representing the capillary bed. We showed that the coupling of the two frameworks is non-trivial since the effects induced by this coupling at microscopic scale have a huge impact on the flow at macroscopic scale. As a consequence, a simple condition of pressure continuity yields large errors in both pressure and flow rate fields. To deal with this issue, we introduced a coupling model that relies on analytical approximations of the pressure field in the vicinity of coupling points. Such a theoretical formulation takes into account the details of the capillary network architecture in the close neighbourhood of the arteriolar and venular endpoints (number, length and diameter of capillaries connected to each coupling point).

By comparison with a complete network approach, we showed that the resulting hybrid approach is accurate in describing the pressure field in various simple test configurations, which involve model capillary lattices with strong topological and morphological differences (6-regular vs. 3-regular networks, networks with uniform or distributed diameters, impact of Virshow-Robin space). We also demonstrated the validity of the hybrid approach when interactions take place between several coupling points (either in the same grid cell or not), or with the domain boundaries. We finally validated this approach on a more complex configuration, combining a few arteriolar and venular trees derived from human anatomical data, with a model capillary network. This last configuration displays 274 coupling points, combining all the geometrical perturbations that typically occur in brain microcirculation (e.g. off-centering, close couplings). Finally, we were able to conclude that the accuracy of the hybrid approach in the large tree-like vessels is similar, on average, to that of a complete network approach.

To go one step further and use anatomical data for the representation of the capillary bed in the future, the model might require several extensions. For instance, the stratification of the vascular density in the depth of the cortex [56] and possible anisotropies [7] may induce strong heterogeneities of capillary networks. To describe this accurately, analytical approximations of the pressure field that capture non-spherical symmetries in the vicinity of couplings may be necessary (see, e.g. [57] for similar problems in petroleum engineering). Moreover, in a perspective of extension to the simulation of mass transfers, it is noteworthy that Darcy’s law is similar to a diffusion equation in pressure. Introducing a hybrid approach for mass transfers will thus involve issues similar to the ones described in the present paper. In the particular case of oxygen transfers, hematocrit heterogeneities induced by the non-proportional distribution of the red blood cells at microvascular bifurcations, i.e. phase separation effect, should also be considered. While this problem has already been investigated in microvascular networks (e.g. [15, 18, 58]), the introduction of such local bi-phasic phenomena in the continuous description of the capillary bed is still a fully open question. Since hematocrit heterogeneities induce non-linear effects, this will likely imply solving the linear system introduced in this paper in an iterative fashion. Thus, the improvement in computational performance will be even more critical for whole-brain computations, not to mention the additional resources needed to solve for the mass transfer problem.

In this perspective, the present hybrid approach has been developed for HPC. With regard to blood flow, this will enable (1) the validation of the hybrid approach by comparison with CN computations for a cortical volume of up to 1 cm^3^ (about ten million of vessels), reaching the computational limit of the CN approach; (2) the simulation of blood flow in much larger volumes—with the ultimate goal of modelling the whole human cortex—via the hybrid approach, assuming that sufficient anatomical data becomes available for arteriolar and venular networks [59]. Such simulations would represent a valuable tool for studying flow variations induced by vascular modifications—vascular occlusions or rarefaction of the capillary vessels observed in Alzheimer’s disease.

## 5 Appendices

### Appendix A Effective properties of the capillary bed

In this appendix, we present how the effective permeability *K*^eff^ and viscosity *μ*^eff^ are calculated in 6- and 3-regular capillary networks displayed in Fig 4. Both coefficients are usually lumped together by considering a single effective resistivity *K*^eff^/*μ*^eff^ of the network. Here, the effective permeability *K*^eff^ represents the permeability of the network with uniform viscosity, while the effective viscosity *μ*^eff^ represents the impact of the Fåhræus-Lindqvist effect (Eq 3).

To compute these effective parameters, the flow is first solved at microscopic-scale in cubic capillary networks of side *L*, using the network approach (Eqs 6 and 7). The results are then averaged at mesoscopic scale and identified to Darcy’s law

$$\begin{array}{c} Q_{L}=\frac{K_{L}^{\text{eff}}L}{\mu_{L}^{\text{eff}}}\Delta_{L}P, \end{array}$$ (35)

where subscript *L* refers to the network side, Δ_*L*_ *P* is the imposed pressure drop and *Q*_*L*_ is the resulting global flow rate. The side *L* of the domain is increased until the effective coefficients do not depend on it anymore, meaning that we have reached the mesoscopic scale at which the capillary bed can be considered as a continuum. The side *L* that corresponds to the convergence of the effective coefficients defines what is commonly called a Representative Elementary Volume (REV) [21, 23]. The characteristics of the capillary networks treated and the final results are summarized in Table 1.

#### Appendix A.1 Uniform capillary diameters

For a uniform capillary diameter *d_cap_* = 8 *μ*m, the viscosity is uniform and computed from the *in* *vivo* viscosity law (Eq 3): *μ*^eff^ ≃ 5.15 × 10^−3^ Pa · s.

In the 6-regular network, we can theoretically derive the corresponding value of *K*^eff^. In a cubic domain of side *L*, this network is indeed similar to *N* × *N* parallel vessels of length *L*, where *N* represents the number of vessels normal to the direction of interest. We have

$$\begin{array}{c} Q_{L}=N^{2}G_{L}\Delta_{L}P, \end{array}$$ (36)

with the conductance corresponding to a vessel of length *L*

$$\begin{array}{c} G_{L}=\frac{\pi d_{cap}^{4}}{128\mu_{L}^{\text{eff}}L}. \end{array}$$ (37)

Noting that *L/N* = *l*_*cap*_ here, we deduce from Eqs 35 and 36 a value of *K*^eff^ that does not depend on *L*

$$\begin{array}{c} K^{\text{eff}}=\frac{\pi d_{cap}^{4}}{128l_{cap}^{2}}. \end{array}$$ (38)

This is valid for *L* > *l*_*cap*_. Here, *K*^eff^ ≃ 4.02 × 10^−14^ m^2^ in each direction, with a REV side equal to *l*_*cap*_.

**Fig 11:**
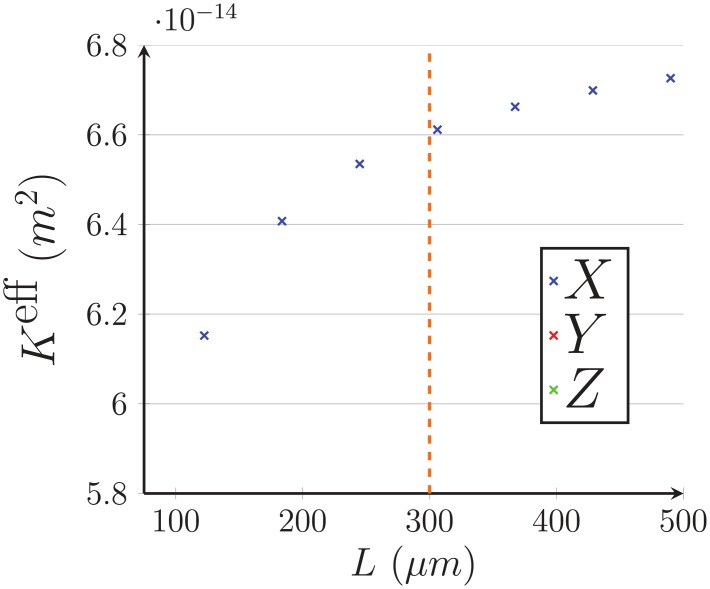
Statistical study for the computation of the effective permeability in a 3-regular capillary network with uniform length (*l_cap_* = 30.62 *μ*m) and diameter *d_cap_* = 8 *μ*m. The variation of the effective permeability *K*^eff^ is plotted in function of the side *L* of the capillary network. The convergence threshold is established at *L* = 300 *μ*m (orange dashed line).

No theoretical formula can be derived to calculate the corresponding permeability *K*^eff^ in the 3-regular network. The REV side and the associated effective parameters are determined by increasing the side of the network *L* until a convergence is observed. As displayed in Fig 11, $K_{L}^{\text{eff}}$ finally reaches a plateau corresponding to the converged effective permeability *K*^eff^. The beginning of the plateau corresponds to the side of the REV. We set the convergence criteria at |*K*(*L*^*n*^) − *K*(*L*^*n*−1^)|/*K*(*L*^*n*^) < 0.01, where *L*^*n*−1^ represents the domain side just below a given side*L*^*n*^. Here, the REV side is about 300 *μ*m and the corresponding permeability value is *K*^eff^ ≃ 6.61 × 10^−14^ m^2^.

#### Appendix A.2 Distributed capillary diameters

**Fig 12:**
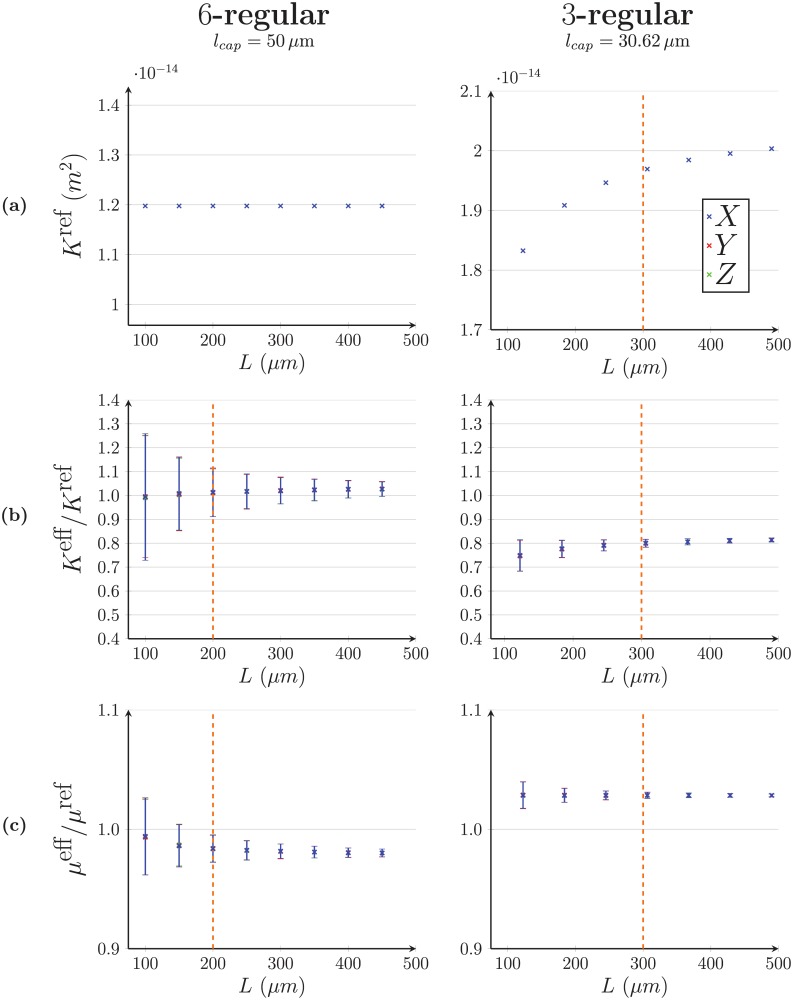
Statistical study for the computation of the effective permeability *K*^eff^ and viscosity *μ*^eff^ in 6- and 3-regular capillary networks with uniform length and distributed diameters. **(a)** First, we derive a reference permeability *K*^ref^ by assuming a uniform diameter, thus a uniform viscosity *μ*^ref^, in the capillaries. The variation of *K*^ref^ is plotted in function of the side *L* of the capillary domain. For 3-regular networks, the convergence threshold is established for *L* = 300 *μ*m (orange dashed line). **(b)** Second, we assume the viscosity to be uniform in the capillaries and derive the effective permeability *K*^eff^ from computations involving 2000 different distributions of diameters. The mean and standard deviation of the ratio *K*^eff^/*K*^ref^ is plotted in function of the side *L* of the capillary domain. For 6-regular networks, the convergence threshold is established for *L* = 200 *μ*m (orange dashed line) **(c)** Finally, we distribute the viscosity in the capillaries and derive the effective viscosity *μ*^eff^ at mesoscopic scale from computations involving the same 2000 different distributions of diameters. The mean and standard deviation of the ratio *μ*^eff^/*μ*^ref^ is plotted in function of the side *L* of the capillary domain.

If the diameter is non-uniform, it follows the Gaussian law detailed in Section 2.6. Since the capillary network is statistically generated—so the diameters distribution follows the Gaussian law, we study the statistical properties of the resulting permeability and viscosity, following the three steps below (results are presented in Fig 12 for both 6- and 3-regular networks):

1. In order to quantify the impact of a diameter distribution on the effective parameters *K*^eff^ and *μ*^eff^, we first compute the reference values *K*^ref^ and *μ*^ref^ in the case of uniform capillary diameters *d*_const_ = 5.91 *μ*m (Fig 12**(a)**), which corresponds to the mean diameter of the Gaussian distribution. From the *in* *vivo* viscosity law, we have *μ*^ref^ ≃ 5.95 × 10^-3^ Pa · s for both networks. The reference permeability *K*^ref^ is then deduced from Eq 35 (Fig 12**(a)**). As expected, *K*^ref^ is constant for the 6-regular network, *K*^ref^ ≃ 1.20 × 10^-14^ m^2^. For 3-regular network, we established the convergence for |*K*(*L*^*n*^) − *K*(*L*^*n*−1^)|/*K*(*L*^*n*^) < 0.01 for *K*^ref^ ≃ 1.9692 × 10^−14^ m^2^ and a REV side of 300 *μ*m.
2. To isolate the impact of geometrical variations on the permeability, we then neglect the viscosity fluctuation and assume the viscosity to be uniform, equal to *μ*^ref^. This means that we define the permeability as depending only on the geometry and topology of the network, independently from the variations of viscosity. We run 2000 computations per side *L*, with 2000 different diameter distributions. For each domain side *L* and diameter distribution *i* in {1, …, 2000}, we compute the corresponding effective permeability $K_{L,i}^{\text{eff}}$ via Eq 35. Mean values and standard deviations of $K_{L}^{\text{eff}}/K^{\text{ref}}$ are presented in Fig 12**(b)**. We establish the convergence for a standard deviation under 0.1. For the 6-regular network, *K*^ref^ ≃ 1.21 × 10^-14^ m^2^ for a REV side of 200 *μ*m. For 3-regular network, *K*^ref^ ≃ 1.58 × 10^-14^ m^2^ for a REV side of 300 *μ*m.
3. To finally capture the variation induced by non-uniform viscosities, we run again the same 2000 computations by letting the *in* *vivo* viscosity law (Eq 3 in the main paper) describe the viscosity fluctuation in each capillary. Now that we know the value of $K_{L,i}^{\text{eff}}$ for each realization *i* in {1, …, 2000}, we can deduce $\mu_{L,i}^{\text{eff}}$ from Eq 35. Mean values and standard deviations of $\mu_{L}^{\text{eff}}/\mu^{\text{ref}}$ are shown in Fig 12**(c)**. For the 6-regular network, *μ*^eff^ ≃ 5.88 × 10^-3^ Pa · s at the REV side of 200 *μ*m. For 3-regular network, *μ*^eff^ ≃ 6.15 × 10^-3^ Pa · s at the REV side of 300 *μ*m.

As expected, we also deduce that the two networks are isotropic because the results overlap in the three directions of space (Figs 11 and 12). Indeed, all networks used in the present paper are periodic in the x, y and z directions, with symmetry planes along the three axis. Thus, according to [21], even if the pressure gradient has not the same principal orientation as the segments of the network, Darcy’s equation still holds. In other words, while segments have preferential orientations at microscopic scale, the medium is isotropic at mesoscopic scale.

### Appendix B Derivation of the reference distance *l*_Γ_ to the coupling

In this section,we detail the derivation the value *l*_Γ_ used in Eq 21 of the main paper. For all capillary vessel *γ* of Ω_*cap*_ that is connected to the coupling point *s*, let *l*_*γ*_ be its length and *π*_*γ*_ the pressure at its endpoint, which is not *s*. We first consider each couple (*π*_*γ*_, *l*_*γ*_), with *γ* inΩ_*cap*_, as references in the analytical solution of Darcy’s law (Eq 12)

$$\begin{array}{c} P\left(r\right)=\pi_{\gamma }-\frac{\mu^{\text{eff}}q_{s}}{4\pi K^{\text{eff}}}(\frac{1}{r}-\frac{1}{l_{\gamma }}). \end{array}$$ (39)

Multiplying by *G*_*sγ*_ and summing over $V_{s}$, we obtain

$$\begin{array}{cc} \sum_{\gamma \in V_{s}}G_{s\gamma }P\left(r_{x}\right) & =\sum_{\gamma \in V_{s}}G_{s\gamma }\pi_{\gamma }-\sum_{\gamma \in V_{s}}G_{s\gamma }\frac{\mu^{\text{eff}}q_{s}}{4\pi K^{\text{eff}}}(\frac{1}{r_{x}}-\frac{1}{l_{\gamma }}), \end{array}$$ (40)

which also can be written as

$$\begin{array}{cc} P(r_{x})= & \frac{1}{\sum_{\gamma \in V_{s}}G_{s\gamma }}\sum_{\gamma \in V_{s}}G_{s\gamma }\pi_{\gamma }\\ & -\frac{\mu^{\text{eff}}q_{s}}{4\pi K^{\text{eff}}}[\frac{1}{r_{x}}-\frac{1}{\sum_{\gamma \in V_{s}}G_{s\gamma }}\sum_{\gamma \in V_{s}}(\frac{G_{s\gamma }}{l_{\gamma }})]. \end{array}$$ (41)

Finally, by identifying *π*_Γ_ and *l*_Γ_, this equation corresponds to Eq 21 with

$$\begin{array}{c} l_{\Gamma }=\frac{\sum_{\gamma \in V_{s}}G_{s\gamma }}{\sum_{\gamma \in V_{s}}\frac{G_{s\gamma }}{l_{\gamma }}}. \end{array}$$ (42)

### Appendix C Matrix structure

**Fig 13:**
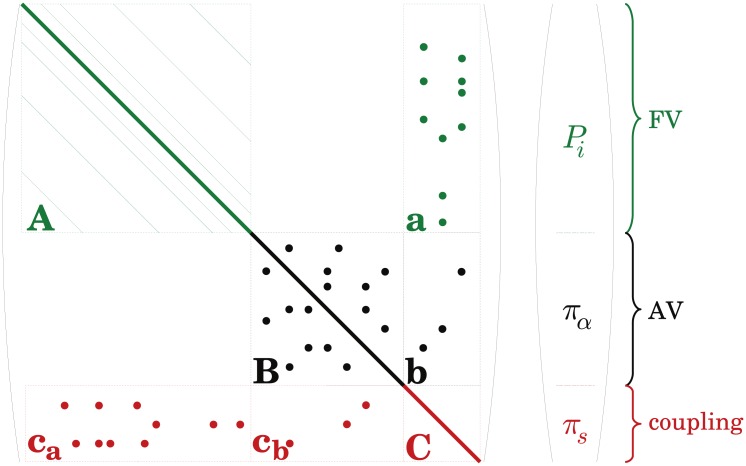
Visualization of the final matrix structure. Using the hybrid approach, we can compute blood flow by solving a single linear system, assembled from the equations describing the finite volume discretization in the continuum (FV, in green), the network approach in the arteriolar and venular trees (AV, in black) and the coupling model at the interface of the two frameworks (coupling, in red). This yields a sparse matrix that is schematized here. Plain lines and dots represent non-zero values. The components (*P*_*i*_)_*i*_, (*π*_*α*_)_*α*_ and (*π*_*s*_)_*s*_ are the unknowns of the linear system, corresponding to FV, AV and coupling parts, respectively.

The matrix describing the linear system for the hybrid approach is a sparse matrix, which is composed of seven parts displayed on Fig 13:

**Part A:** the finite volume formulation (Eq 29) yields a symmetric sparse block of size (number of cells)^2^. Each row *i* contains 7 components: one diagonal value associated to the cell *i* and 6 off-diagonal values associated to its neighbours;
**Part a:** the finite volume formulation applied to the coupled cells yields a supplementary off-diagonal block of size (number of cells×number of couplings), where the components correspond to the distributed impact of the couplings and the relationship with the associated coupled vertices;
**Part B:** the network approach (Eqs 6 and 7) yields another sparse block of size (number of inner vertices)^2^. Each row *α* contains $1+\text{Card}(N_{\alpha })$ components, where $\text{Card}(N_{\alpha })$ is the number of neighbouring vertices of the inner vertex *α*: one diagonal value associated to the inner vertex *α* and $\text{Card}(N_{\alpha })$ off-diagonal values associated to its neighbours;
**Part b:** some inner vertices of the network approach are connected to coupling points instead of boundary nodes, yielding a supplementary off-diagonal block of size (number of inner vertices×number of couplings), where the components correspond to the associated coupled vertices;
**Part C:** the coupling part (Eq 25) yields a simple diagonal block of size (number of couplings)^2^. Each row *s* corresponds to a new unknown, which is associated to the coupled vertex *s*;
**Part c_a_:** the coupling part is completed by an off-diagonal block of size (number of couplings×number of cells). The components correspond to the connection of each coupled vertex to the FV cells where the coupling condition has been distributed (as in S1 Fig of the Supporting Information);
**Part c_b_:** the coupling part is also completed by an off-diagonal block of size (number of couplings×number of inner vertices). The components correspond to the connection of each coupled vertex to its unique neighbouring vertex in the network part.

The right-hand side, which describes the boundary conditions imposed at the inlets and outlets of the arteriolar and venular trees, is finally very sparse.

### Appendix D Nomenclature

In this section, a detailed nomenclature is provided about the description of the domains used in the main paper. The domains are sorted according to the elements that they contain: network domains contain vessels, finite volume domains contain discretization cells, and continuous domains are subdomains of $R^{3}$. Each of the domain detailed below is assigned to one of the following classes: main structural component, domain involved in the development of the coupling model, validity domains of the analytical approximations, and domain dedicated to the computation of errors.

#### Appendix D.1 Network domains

- Ω_*AV*_ is the set of network vessels in the arteriolar and venular trees (main structural component);
- *s* is a coupling point, at the interface between Ω_*AV*_ and Ω_*FV*_ (main structural component);
- Ω_*cap*_ is the set of capillary vessels connected to a coupling point (main structural component).

#### Appendix D.2 Finite volume domains

- Ω_*FV*_ is the set of cells used to discretize the continuum (main structural component);
- $\Omega_{FV}^{\varepsilon }$ is a reduction of Ω_*FV*_, which excludes the sets $\Omega_{FV,neigh}^{s}$ (computation of errors);
- $\Omega_{FV,neigh}^{s}$ is the set of cells that contains the cell coupled to the coupling point *s* and its neighbours, including the diagonal neighbours (validity of the analytical approximations);
- ${\bar{\Omega }}_{FV,neigh}^{s}$ is a reduction of $\Omega_{FV,neigh}^{s}$, which excludes the coupled cell (development of the coupling model);
- ${\bar{\Omega }}_{sph}^{s}$ is the set of cells that share a face with $\Omega_{FV,neigh}^{s}$ (development of the coupling model);
- $\Omega_{sph}^{s,\varepsilon }$ is an extension of ${\bar{\Omega }}_{sph}^{s}$, which further includes the cells sharing a corner with $\Omega_{FV,neigh}^{s}$ (computation of errors).

#### Appendix D.3 Continuous domain

- Ω_*lin*,*s*_ is a spherical subdomain of $R^{3}$ of radius *l*_Γ_, centered in the coupling point *s* (validity of the linear approximation);
- $\Omega_{sph}^{s}$ is a subdomain of $R^{3}$ that corresponds to $\Omega_{FV,neigh}^{s}\cup {\bar{\Omega }}_{sph}^{s}$ (validity of the spherical approximation).

## Supporting information

S1 Fig2D schematic diagrams of the distribution of the coupling condition for off-centered coupling point.To take into account the off-centering of a given coupling point *s* (red dot), the corresponding flow rate *q*_*s*_ is distributed among each cell *i* of $\Omega_{FV,neigh}^{s}$ (yellow hatching) according to the partition coefficient *τ*_*i*_ = *v*_*i*_/*h*^3^, where *v*_*i*_ is the intersection between the cell *i* and a fictitious mesh cell centered on the coupling point (black square), and *h*^3^ the volume of a cubic discretization cell.(EPS)

S2 Fig Comparison of hybrid and reference CN approaches for the Baseline configuration displayed in Fig 6i(a). Here, a simple condition of pressure continuity is imposed at the interface between the arteriolar vessel and the continuum. **(a)** *Global pressure errors.* Global pressure errors are computed in the four domains of interest defined in 3**(d)**. The errors are normalized by the global pressure drop *δP*. **(b)** *Global flow rate errors.* The flow rate errors are computed in the same four domains of interest. The errors are normalized by the global incoming flow rate *q*_*A*_.
(EPS)

S3 Fig Comparison of the hybrid approach with reference complete network computations. In complete network version, arteriolar and venular trees are connected to 6- or 3-regular capillary networks. In the hybrid approach, they plunge into an equivalent porous medium which is discretized in finite volume cells of size *h*. **(a)** In configuration **i**, a single coupling is imposed at the center of the coupled cell. We then vary *h*/*l*_*cap*_ ratio where *l_cap_* = 50 *μ*m represents the characteristic length of a capillary vessel. In the following configurations, the same study has been led by taking into account: **ii**. the Virshow-Robin space (non-vascularization around arteriole [7]); **iii**. the diameters distribution; and **iv**. the physiological 3-regularity of the capillary network. **(b)** For each configuration, pressure errors are computed in four domains of interest (Fig 3**(e)** of the main paper). The errors are normalized by the global pressure drop *δP*. **(c)** The maps of the local pressure errors are displayed for ratios of interest indicated by orange dotted lines in above graphs. **(d)** The flow rate errors are computed in the same four domains of interest. The errors are normalized by the global incoming flow rate *q*_*A*_.
(EPS)

S4 Fig Comparison of pressure profiles computed via hybrid and CN approaches, the hybrid approach being associated to a simple condition of pressure continuity at the coupling point. The pressure profile is plotted for a single centered coupling, as in Fig 6i(a) in the main paper. The reference pressure field from CN approach is represented by orange crosses and green steps represent cell pressures of the FV representation. The pressures *π*_H_ and *π*_CN_ at the endpoint of the coupled vessel, for both approaches, are indicated by tick red and orange dashes, respectively. Here *π*_ref_ = 5000Pa is a reference pressure value used for nondimensionalization.
(EPS)

S5 Fig Global pressure and flow rate errors for multiple couplings occuring in the same FV cell. From 2 to 10 arteriolar and venular vessels are randomly coupled to a 6-regular capillary network, in such a way that they are located in the same FV cell in the FV domain of the equivalent hybrid configuration. According to physiological statistics (Fig 2**(b)**), a minimal distance of 100 *μ*m is imposed between the arteriolar inlets and venular outlets. **(a)** *Global pressure errors.* Mean and standard deviations of the pressure errors are computed in four domains of interest defined in Fig 3**(e)** of the main paper, for 100 realisations. The errors are normalized by the global pressure drop *δP*. **(b)** *Global flow rate errors.* Mean and standard deviations of the flow rate errors are computed in the same four domains of interest. The errors are normalized by the global incoming flow rate *q*_*A*_.
(EPS)

S6 Fig Comparison between the hybrid and complete network approaches in a larger realistic configuration. Pressure and flow rate values are compared in a log-log plot. Black crosses refer to the hybrid approach using our coupling model. Gray crosses refer to the hybrid approach using a simple condition of pressure continuity at the interface between arteriolar, venular vessels and the continuum. Here, *π*_ref_ = 5 000 Pa and *q*_ref_ = 5 × 10^−12^ m^3^ · s^−1^ are reference pressure and flow rate values used for nondimensionalization.
(EPS)

S7 Fig Scale differences between oil reservoirs and the human cortex. Three characteristic length scales are presented for both petroleum and cerebral applications: the pore size of the porous medium (oil reservoir or capillary bed), the diameter of the coupled component (wellbore or arteriolar/venular vessel) and the typical size of a discretization cell in the finite volume representation of the continuum. Each length is normalized by the typical size of a mesh cell in the corresponding application.
(EPS)
